# Identification of Galectin-9 (Gal-9) as a B7-H4 binding partner and characterization of their glycosylation-dependent interaction that modulates T cell signaling within a multi-ligand/receptor network

**DOI:** 10.1371/journal.pone.0355964

**Published:** 2026-09-18

**Authors:** Ravear Zhiqiang Wang, Fang Yang, Jianhua Sui

**Affiliations:** 1 School of Life Sciences, Beijing Normal University, Beijing, China; 2 National Institute of Biological Sciences, Beijing, China; 3 Tsinghua Institute of Multidisciplinary Biomedical Research, Tsinghua University, Beijing, China; Sungkyunkwan University - Suwon Campus: Sungkyunkwan University - Natural Sciences Campus, KOREA, REPUBLIC OF

## Abstract

B7-H4, a member of the B7 family, is broadly expressed on various cancer cells and has been implicated in negative immune regulation, particularly in suppressing anti-tumor immunity. However, its receptor and the mechanisms underlying its immunosuppression remain poorly understood. Here, we identify Galectin-9 (Gal-9) as a binding partner of B7-H4 and investigate its role in modulating T cell responses. We show that glycosylation within the IgC domain of B7-H4 is required for Gal-9 binding, while the N-terminal carbohydrate recognition domain (N-CRD) of Gal-9—specifically residue R65—is essential for its binding with B7-H4. In addition, several other B7 family members (B7.1, B7.2, B7-H2, and B7-DC) and immune cell surface receptors (CD28, 2B4, CD226, and SLAMF1) also bind to Gal-9 at levels comparable to those observed with B7-H4 or TIM-3. *In vitro* functional assays revealed that B7-H4 inhibits Gal-9-induced activation of CD28 downstream signaling and reduces Gal-9-mediated T cell death. *In vivo*, Gal-9 deficiency in mice resulted in an increased proportion of splenic CD4^+^ T cells, whereas B7-H4 deficiency produced no detectable phenotype. Moreover, B7-H4 and Gal-9 double-knockout mice showed no additive phenotype compared with Gal-9 single-knockout mice, and tumor growth following tumor cell challenge was unaffected in all three knockout models. Collectively, these findings indicate that B7-H4, Gal-9, other B7 family members, and T cell surface immune receptors form a complex regulatory network that modulates T cell activity and anti-tumor responses, with no single component exerting a dominant effect. This study provides a detailed molecular characterization of the B7-H4–Gal-9 interaction and uncovers additional Gal-9 binding partners, offering insights into the finely tuned immune regulation mediated by the B7 family.

## Introduction

B7-H4 (B7 homolog 4, also known as B7S1, B7x, or VTCN1) is an orphan immune checkpoint ligand belonging to the B7 family. It has been proposed that B7-H4 negatively regulates T cell immune response through multiple mechanisms, including the suppression of T cell proliferation in response to antigen stimulation [1], the reduction of key cytokines, such as IL-2 and IFN-γ [2], the induction of T cell anergy [3], and the protection of normal tissues from T cell-mediated destruction [4]. B7-H4 is frequently overexpressed in various cancers and tumor-associated macrophages, where it contributes to the suppression of anti-tumor immunity [5–9]. These findings underscore the role of B7-H4 in regulating immune responses in both physiological and pathological contexts. However, the identity of its receptor and the precise molecular mechanisms through which it inhibits immune responses remain unclear [10].

A previous study reported that B7-H4 interacts with activated T lymphocytes from wild type (WT) mice, but not with T cells from B and T lymphocyte attenuator (BTLA)-deficient mice [11], indirectly suggesting that BTLA could be a potential receptor for B7-H4. However, subsequent studies have shown that BTLA is unlikely to function as a receptor for B7-H4, and herpesvirus entry mediator (HVEM) has been identified as the ligand for BTLA [12,13]. Another study proposed an indirect mechanism in which B7-H4 associates with soluble Sema3a, engaging a Nrp-1/Plexin A4 complex on regulatory T cells [14]. Nevertheless, Sema3a itself does not serve as a direct receptor for B7-H4. Intriguingly, other research indicates that tumor-infiltrating CD8^+^ T cells may express a putative receptor for B7-H4, as antigen-presenting cells (APCs) expressing B7-H4 suppress the effector functions of CD8^+^ T cells [15]. These findings highlight the complexity of B7-H4-dependent immune regulation and underscore the need for further investigation.

Galectin-9 (Gal-9), a member of the galectin family, plays a role in regulating human immune responses through multiple signaling pathways. Gal-9, which is expressed in T cells, B cells, and dendritic cells [16–18], participates in the intricate network of immune cell activation, proliferation, and autophagy [19]. Aberrant expression of Gal-9 has been detected in various hematological malignancies (e.g., lymphoma, leukemia) and solid tumors (e.g., pancreatic cancer, breast cancer, hepatocellular carcinoma) [20–22]. Through its interaction with immune checkpoint receptors, Gal-9 displays multifaceted roles in regulating immune responses. Gal-9 interacts with TIM-3, leading to the induction of Th1 cell death [23]. The C-terminal carbohydrate recognition domain (C-CRD) of Gal-9 binds with PD-1 in cis on T cells, forming a TIM-3/Gal-9/PD-1 complex and thus reducing TIM-3-dependent T cell death [24]. Gal-9 facilitates Kupffer cell-dependent phagocytosis against cancer cells through its interaction with ERMAP and Dectin-2 [25]. Gal-9 binds with the CD44 immune receptor, thereby enhancing the stability of regulatory T cells and promoting neutrophil adhesion [26,27]. However, the requirement for Gal-9 engagement via B7 family ligands and CD28 family receptors to modulate T cell-mediated immune responses remains largely unknown.

Herein, we identify Galectin-9 (Gal-9) as a previously unknown binding partner for B7-H4 using an immunoprecipitation (IP) coupled with mass spectrometry (MS) approach, where recombinant B7-H4 protein served as a bait protein, while peritoneal immune cells—harvested from mice adoptively transferred with EG7 tumor cells and OVA-specific T cells (OT-1 T cells)—were used as target cells. Further characterization of the interaction between B7-H4 and Gal-9 revealed that glycosylation in the IgC domain of B7-H4 is required for its binding to Gal-9; while the N-terminal carbohydrate recognition domain (N-CRD) and the R65 residue within the N-CRD of Gal-9 are essential for this interaction. Notably, we also discovered that Gal-9 binds several previously unrecognized immune receptors on T cells, including CD28, and other B7 family members. Soluble glycosylated B7-H4 attenuates Gal-9’s dual activities: the stimulation of CD28/AKT signaling and the induction of T cell death. Furthermore, a comparison of the splenic immune cell compositions revealed that the pattern and extent of the increased proportion of CD4^+^ T cells in Gal-9 knockout (KO) mice was similar to that in B7-H4 and Gal-9 double knockout (DKO) (B7-H4/Gal-9 DKO) mice, whereas B7-H4 KO mice showed no such increase, indicating that B7-H4 KO alone is insufficient to alter Gal-9’s activity in this context *in vivo*, consistent with the observation that multiple B7 family members and immune receptors on T cells interact with Gal-9. Together, our findings suggest that B7-H4, other B7 family members, and T cell surface immune receptors interact with Gal-9 within a complex regulatory network that finely modulates T cell activity through multiple overlapping pathways.

## Materials and Methods

### Cell lines and primary immune cells

Jurkat, MOLT-4, 293T, 4T1, MC38, and EG7 cells were from the Cell Bank of Type Culture Collection (Chinese Academy of Sciences) or the American Type Culture Collection (ATCC). Freestyle 293F cells were from Thermo Fisher Scientific. *MGAT1* (encoding GNT-I) gene KO 293F cells were previously generated in our laboratory [28]. Jurkat and MOLT-4 T cells were cultured in RPMI-1640 medium supplemented with 10% FBS. 293T cells were cultured in DMEM medium containing 10% FBS. 293F cells were cultured in SMM 293-TII medium. 4T1 cells were cultured in ATCC-formulated RPMI-1640 medium supplemented with 10% FBS. MC38 cells were cultured in DMEM supplemented with 10% FBS. EG7 cells were cultured in ATCC-formulated RPMI-1640 supplemented with 0.05 mM 2-mercaptoethanol, 10% FBS, and 0.4 mg/mL of G418. Primary human T cells were isolated from frozen human PBMCs (Ori Biotech, FPB003F-C) using the EasySep™ Human T Cell Enrichment Kit (Miltenyi Biotec) and maintained in RPMI-1640 without serum. Mouse splenic immune cells from Gal-9 KO mice were isolated using Red Blood Cell Lysis Buffer (0.15 M NH_4_Cl, 10 mM KHCO_3_, 0.1 mM Na_2_EDTA).

### Stable cell line generation

Jurkat-NFAT-Luc stable reporter cell line was generated by transfecting Jurkat T cells with a plasmid carrying nuclear factor of activated T cells (NFAT)-RE-luciferase reporter encoding gene (luciferase gene under the control of an NFAT-response element) following the instruction of Amaxa Cell Line Nucleofector Kit V. The transfected cells were selected with 7.5 µg/mL antibiotics Blasticidin S (InvivoGen, ant-bl-05) and subjected to limiting dilution cloning to obtain single stable cell clone. To assess NFAT activation, the selected stable clones were incubated with test agents, and NFAT signaling activity was quantified by measuring luminescence using the Bright-Glo™ Luciferase Assay System Kit (Promega, E2620).

MC38-mB7-H4 stable cells were established by transducing MC38 cells with lentiviruses expressing the full-length mB7-H4 protein. The lentiviruses were produced by transient transfection of 293T cells with the pLKO.1-VSV-G packaging plasmids. Lentivirus-transduced MC38 cells were selected with 5 µg/mL puromycin (Amresco, J593-25MG) and subsequently sorted using flow cytometry to isolate single-cell clones stably expressing cell-surface mB7-H4. A candidate MC38-mB7-H4 stable cell clone, which exhibited a proliferation rate comparable to that of parental MC38 cells, was selected for subsequent tumor inoculation studies in mice.

### Antibodies and reagents

Antibodies and reagents used for flow cytometry analysis were primarily obtained from BioLegend unless otherwise indicated: FITC anti-mouse CD45 (clone 30-F11), FITC anti-mouse CD45.1 (clone A20), APC-Cy7 anti-mouse CD45.2 (BD Biosciences, clone 104), PE-Cy7 anti-mouse CD3 (clone 17A2), PerCP-Cy5.5 anti-mouse CD19 (clone 6D5), BV786 anti-mouse CD11b (BD Biosciences, clone M1/70), BV605 anti-mouse CD4 (clone GK1.5), BV421 anti-mouse NK1.1 (clone PK136), APC anti-mouse CD8 (clone 53-6.7), LIVE/DEAD Fixable Dead Cell Stain (Thermo Fisher Scientific, L34976), PerCP anti-human CD3 (clone UCHT1), BV605 anti-human CD4 (clone OKT4), APC anti-human CD8 (clone HIT8a), PE-Cy7 anti-human CD14 (clone HCD14), FITC anti-human CD19 (clone HIB19), BV421 anti-human CD56 (clone HCD56).

Antibodies and reagents used for western blotting were primarily obtained from ABclonal unless otherwise indicated: Phospho-CD28 (pCD28; Y191) Rabbit mAb (Cell Signaling Technology, 16399), CD28 Rabbit mAb (A20346), Phospho-Akt (pAKT; S473) Rabbit mAb (AP1208), Pan-Akt Rabbit mAb (A18675), HRP-conjugated β-Actin Rabbit mAb (AC028), Protease/Phosphatase Inhibitor Cocktail (Cell Signaling Technology, 5872), and Lactose (LABLEAD BIOTECH, 5989-81-1).

Human Galectin-9 (Gal-9) protein was purchased from R&D Systems (Cat. 2045-GA).

### Protein expression, purification, and PNGase F deglycosylation

The mB7-H4-Bio protein was expressed by co-transfecting 293F cells using PEI-MAX with two plasmids: an expression plasmid encoding mB7-H4 ECD-His_6_-Avi, the mouse B7-H4 extracellular domain (ECD; UniProt Q7TSP5, residues 1–261) fused to a C-terminal His₆-Avi tag (GLNDIFEAQKIEWHE), and a BirA biotin ligase-expressing plasmid. Five days post-transfection, the culture supernatant was harvested and purified by Ni-NTA Agarose affinity chromatography (QIAGEN, 30230). Similarly, human Sema3a (UniProt Q14563, residues 1–771) with a C-terminal His₆ tag was expressed in 293F cells and purified.

The mB7-H4-Fc protein was expressed using an expression plasmid constructed with the cDNA encoding the mB7-H4 ectodomains, comprising the IgV and IgC domains (UniProt Q7TSP5, residues 32–261), fused to the human IgG1 Fc region. Similarly, expression plasmids for hB7-H4-Fc, hB7-H4 IgV-Fc, and hB7-H4 IgC-Fc proteins were constructed using the cDNAs encoding the hB7-H4 ectodomain (UniProt Q7Z7D3-1, residues 32–259), the IgV domain (UniProt Q7Z7D3-1, residues 36–148), and the IgC domain (UniProt Q7Z7D3-1, residues 153–259) respectively, fused to the mouse IgG2a Fc region. 293F cells were transiently transfected with these expression plasmids using PEI-MAX transfection reagents. Five days post-transfection, the culture supernatant was harvested and purified using Protein A beads. Protein concentrations were determined using a Nanodrop spectrophotometer. The de-glycosylated hB7-H4-Fc protein (B7-H4^MGAT1-^) was produced by transient transfection of *MGAT1* KO 293F cells with the hB7-H4-Fc plasmid, and purified using Protein A affinity chromatography, similar to the hB7-H4-Fc described above.

The recombinant proteins (Fc tag, mouse IgG2a isotype) of multiple human immune receptors, including 2B4 (UniProt Q9BZW8-2, residues 1–224), PD-1 (UniProt Q15116, residues 1–170), TIM-3 (UniProt Q8TDQ0-1, residues 1–202), CD226 (UniProt Q15762, residues 1–254), SLAMF1 (UniProt Q13291-1, residues 1–237), CD28 (UniProt P10747-1, residues 1–152), BTLA (UniProt Q7Z6A9-1, residues1–156), ICOS (UniProt Q9Y6W8-1, residues 1–140), TIGIT (UniProt Q495A1-1, residues 1–141), CD305 (UniProt Q6GTX8-1, residues 1–165), CD160 (UniProt O95971-1, residues 1–159), CTLA-4 (UniProt P16410-1, residues 1–161), CD2 (UniProt P06729, residues 1–209), B7.1 (UniProt P33681-1, residues 1–242), B7.2 (UniProt P42081-1, residues 1–247), B7-DC (UniProt Q9BQ51-1, residues 1–220), B7-H1 (UniProt Q9NZQ7-1, residues 1–238), B7-H2 (UniProt O75144-1, residues 1–256), and B7-H3 (UniProt Q5ZPR3-2, residues 1–248) were expressed and purified, similar to the hB7-H4-Fc described above.

To generate site-directed mutants of human B7-H4, PCR primers targeting specific mutation sites were designed using TAKARA Primer design tools and employed to amplify DNA fragments in accordance with the manual of High-fidelity DNA polymerase KOD-Plus (TOYOBO). Molecular cloning was carried out according to the manual of In-Fusion HD Cloning Kit (Takara). These B7-H4 mutants (Fc tag, mouse IgG2a isotype) were expressed and purified as described above.

For removing the N-linked glycosylation modifications and preparing of de-glycosylated hB7-H4-Fc, 10 µg of hB7-H4-Fc and 2 µL of PNGase F (New England Biolabs, P0704S) were mixed and then incubated for 24 h at 37 °C under non-denaturing reaction conditions. An untreated control (10 μg hB7-H4-Fc without PNGase F) was processed in parallel. To prepare the denaturing reaction using PNGase F, hB7-H4-Fc was first pre-denatured at 100 °C for 10 min. A mixture of 10 µg of pre-denatured protein, 2 µL of GlycoBuffer 2 (New England Biolabs), 2 µL of 10% NP-40, 6 µL of H_2_O, and 1 µL of PNGase F, was then incubated for 1 h at 37 °C. The treated protein samples were then subjected to SDS-PAGE electrophoresis and Coomassie blue staining for confirming the de-glycosylation.

### Co-immunoprecipitation-mass spectrometry (Co-IP-MS)

In the Co-IP for MS, 50 µL of M-280 streptavidin magnetic beads were incubated with 1 mL cell lysate supernatant supplemented with or without 4 µg mB7-H4-Bio bait protein overnight at 4 °C. Following incubation, the beads were washed four times with PBST buffer (PBS containing 0.05% Tween-20). The washed M-280 magnetic beads carrying the precipitated proteins were subjected to sodium dodecyl sulfate-polyacrylamide gel electrophoresis (SDS-PAGE). Silver staining analyses were conducted to identify candidate binding proteins for mB7-H4. Protein bands of interest, which were observed in the experimental lane with mB7-H4-Bio but absent in the control lane (without the bait protein), were excised and subjected to LC-MS/MS analysis at the Proteomics Facility of National Institute of Biological Sciences, Beijing (NIBS).

### Western blotting

To examine the bait protein mB7-H4-Bio used in the Co-IP-MS, the precipitated proteins were resolved by SDS-PAGE and transferred to a nitrocellulose (NC) membrane using a wet transfer method. The NC membrane was blocked with 3% (w/v) non-fat milk in PBST for 1 h at room temperature, followed by incubation with HRP-Streptavidin (Thermo Fisher Scientific, 21130) in blocking buffer for 1 h at 37 °C. After five washes with PBST, the membrane was immersed into ECL substrate for 5 min. All blots on the x-ray films were processed inside the darkroom.

For cell lysate samples, the whole-cell lysates were mixed with SDS buffer at room temperature for 30 min, heated at 100 °C for 10 min, and centrifuged at 12,000 g for 10 min. Supernatants (4–8 µL) were separated by SDS-PAGE and transferred onto NC membranes. Membranes were incubated overnight at 4 °C using the primary antibodies described above.

### Co-IP-Western blotting of cell-surface CD28 or B7-H4 interaction with Gal-9

To assess the interaction between cell-surface CD28 and Gal-9, Jurkat T cells were lysed in Western/IP lysis buffer (Beyotime, P0013) supplemented with 1 mM PMSF. Cell lysates were incubated under the indicated conditions: with or without 4 µg/mL Gal-9 in the presence or absence of 200 mM lactose for 2 h at 4 °C, followed by IP using an anti-CD28 rabbit monoclonal antibody (Abcam, ab243228) and Protein A magnetic beads (GenScript, L00695) overnight at 4 °C. The beads were washed three times with TBST, and bound proteins were eluted with glycine-HCl buffer (pH 2.5). Eluted proteins were mixed with SDS sample buffer, heated at 100 °C for 10 min, separated by SDS-PAGE, and transferred onto NC membranes using a wet transfer system. Western blotting was then performed as described above using an anti-CD28 rabbit monoclonal antibody (ABclonal, A20346), different from that used for immunoprecipitation, and an anti-Gal-9 mouse monoclonal antibody (R&D Systems, MAB20455) as primary antibodies, followed by appropriate HRP-conjugated secondary antibodies: HRP-anti-Rabbit IgG antibody (Jackson ImmunoResearch, 111-035-003), HRP-anti-mouse IgG antibody (Jackson ImmunoResearch, 115-035-003), and HRP-anti-GAPDH antibody (ABclonal, AC035).

The interaction between cell-surface B7-H4 and Gal-9 was examined using a similar procedure. Human B7-H4 (UniProt Q7Z7D3-1, residues 1−282) with a C-terminal His_6_ tag was transiently expressed in 293T cells using Lipofectamine 2000 (Thermo Fisher Scientific, 11668030). After 24 h of transfection, whole-cell lysates (WCL) from B7-H4-expressing 293T cells (293T-B7-H4) were incubated with or without 4 µg/mL Gal-9, in the presence or absence of 200 mM lactose for 2 h at 4 °C, followed by immunoprecipitation with an anti-B7-H4 rabbit monoclonal antibody (Cell Signaling Technology, 14572) and Protein A magnetic beads overnight at 4 °C. Bound proteins were then eluted and analyzed by western blotting using an anti-B7-H4 rabbit monoclonal antibody (Abcam, ab252438), different from the antibody used for immunoprecipitation, and the anti-Gal-9 mouse monoclonal antibody (R&D Systems, MAB20455) as primary antibodies, followed by appropriate HRP-conjugated secondary antibodies: HRP-anti-Rabbit IgG antibody (Jackson ImmunoResearch, 111-035-003), HRP-anti-mouse IgG antibody (Jackson ImmunoResearch, 115-035-003), and HRP-anti-GAPDH antibody (ABclonal, AC035).

### Surface plasmon resonance (SPR)

The binding kinetics between hB7-H4-Fc protein, its variants, and other Fc-tagged proteins to human Gal-9 were analyzed using SPR on a Biacore T200 instrument (Biacore, GE Healthcare). Protein A/G was immobilized on a CM5 sensor chip using an amine-coupling kit (GE Healthcare). hB7-H4-Fc or its variant proteins (1 μg/mL) were captured on the sensor chip, followed by the flow of two-fold serially diluted Gal-9 starting at 33 nM. Binding kinetics were analyzed using Biacore T200 evaluation software to calculate the association rate constant (ka), dissociation rate constant (kd), and equilibrium dissociation constant (KD).

### Jurkat-NFAT reporter bioassay

Jurkat-NFAT-Luc reporter stable cells were mixed with hB7-H4-Fc or Gal-9 proteins at indicated concentrations or mixed with 293F-B7-H4 cells (293F cells transiently expressing full-length hB7-H4), and incubated for 3 h at 37 °C in a 96-well plate. Cells were centrifuged (400 g, 5 min), resuspended in 70 μL of Glo Lysis Buffer (Promega, E2661), and incubated for 5 min. 50 µL of the cell lysate was transferred into a 96-well white bottom microplate and mixed with 50 µL of Bright-Glo Luciferase Assay Substrate (Promega, E2620). NFAT-mediated luminescence was measured using GloMax Navigator System (Promega).

### Gal-9-mediated CD28/AKT activation

To examine Gal-9-mediated stimulation of pCD28 signaling activity in the presence of plate-bound B7.1, serum-starved Jurkat T cells (1.0 × 10^5^ cells per well) were treated with Gal-9 (3–7 µg/mL) in a 96-well plate pre-coated with 10 µg/mL hB7.1-Fc for 2 h at 37 °C. Following the incubation for 30 min at 37 °C, the plate was centrifuged at 400 g for 5 min to harvest cell pellets. The resulting cell pellets were lysed in RIPA buffer containing protease and phosphatase inhibitors for 30 min at 4 °C. The cell lysates were analyzed by western blotting as mentioned above. To assess the regulation of Gal-9-mediated stimulation of CD28 signaling by glycosylated B7-H4, serum-starved T cells (Jurkat, MOLT-4, or primary T cells; 1.0 × 10^5^ cells per well) were treated with 8 µg/mL Gal-9 for 30 min at 37 °C in the presence or absence of 25 µg/mL glycosylated hB7-H4-Fc, hB7-H4 IgV-Fc, or de-glycosylated B7-H4^MGAT1-^ fusion proteins. After centrifugation at 400 g for 5 min to collect cell pellets, the cell lysates were prepared and analyzed by western blotting as described above.

### Cell viability assay

Serum-starved MOLT-4, Jurkat, or primary T cells (1.0 × 10^5^ cells/sample) were filtered through 40 µm strainers and treated with 8 µg/mL Gal-9 in the presence or absence of 25 µg/mL glycosylated hB7-H4-Fc or de-glycosylated B7-H4^MGAT1-^ for 30 min. Following the treatment, 7-aminoactinomycin D (7-AAD) was added to the T cells and incubated for 5 min. Flow cytometry data were acquired using the BD Accuri C6 instrument and analyzed using Flowjo (v10.6.2). The analysis focused on determining the proportion of live T cells (7-AAD negative) within the parent gate. To examine the regulation of T cell viability by immobilized B7-H4 proteins, glycosylated B7-H4 or deglycosylated B7-H4^MGAT1-^ (8 µg/mL) were immobilized overnight on Protein A beads (Smart-Lifesciences, SA023005). The next day, 1.0 × 10^5^ of serum-starved MOLT-4 T cells were incubated for 30 min at 37 °C with Gal-9 (6 µg/mL) and either iB7-H4- or iB7-H4^MGAT1-^-immobilized beads. Cells were then stained by 7-AAD for 5 min and analyzed by flow cytometry as previously described.

To assess Gal-9 effects on PBMC viability, human PBMCs (5 × 10^5^/well) were seeded in a 96-well plate and treated with or without 8 µg/mL Gal-9 for 30 min. After washing twice with PBS, cells were stained with LIVE/DEAD^TM^ Fixable Near-IR dye (Thermo Fisher Scientific, L34975) for 30 min at 4 °C. Following this, the cells were washed twice with 0.5% BSA/PBS, and incubated with primary antibodies targeting human immune lineages for 15 min at 4 °C. Following two additional washes, cells were resuspended in 300 µL of PBS for flow cytometry data acquisition on a BD LSRFortessa™. Data were analyzed using FlowJo (v10.6.2). Cell viability of CD3^+^ T cell and CD19^+^ B cell proportions was quantified using GraphPad Prism (v9.2.0).

### Flow cytometry

For examining the binding of mB7-H4-Fc to host CD45.1^+^ immune cells or CD45.1^-^ OT-1 T cells harvested from the peritoneal cavity of adoptive-transfer-model mice, the harvested immune cells were stained by an amino-reactive dead cell dye (Thermo Fisher Scientific, L34975) for 30 min at 4 °C. After two washes with 0.5% BSA/PBS, the immune cells were pre-blocked with anti-mouse CD16/32 antibody (Biolegend, 101301) and 4% (v/v) mouse serum for 10 min at room temperature, and subsequently incubated with or without mB7-H4-Fc for 30 min at 4 °C. Following two additional washes, cells were stained by: PE anti-human Fc antibody (Thermo Fisher Scientific, 12-4998-82), APC-Cy7 anti-mouse CD45.2 (BD Biosciences, clone 104), FITC anti-mouse CD45 (BioLegend, clone 30-F11), or FITC anti-mouse CD45.1 (BioLegend, clone A20) for 15 min at 4 °C. Following another two washes, these cells were resuspended in 300 µL of PBS for flow cytometry data acquisition on a BD LSRFortessa^TM^. All flow cytometry data were analyzed with Flowjo (v10.6.2).

The interaction between Fc-tagged proteins (hB7-H4-Fc variants/others) and cell-expressed Gal-9 or its variants were analyzed using transiently transfected 293T cells. The 293T cells were transfected to express either the full-length Gal-9 (UniProt O00182-1, residues 1–355, including both the N-CRD and C-CRD domains) or its truncation/mutation variants, each fused with EGFP. The transfected 293T cells were fixed and permeabilized following the instructions of the Fixation/Permeabilization Kit (BD). Subsequently, these cells were incubated with 10 µg/mL test proteins for 30 min at 4 °C. After washing twice with Perm/Wash Buffer (BD), the cells were stained with Alexa Fluor 647 anti-mouse Fc antibody (Thermo Fisher Scientific, 1839633) for 20 min at 4 °C. Following two additional washes, the cells were resuspended in PBS and analyzed by flow cytometry. Flow cytometry data were acquired and analyzed as described above. The interaction of cell-expressed Gal-9 with multiple human immune receptors (hPD-1-Fc, hTIM-3-Fc, hCD28-Fc, h2B4-Fc, hSLAMF1-Fc, hBTLA-Fc, hICOS-Fc, hTIGIT-Fc, hCD305-Fc, hCD160-Fc, hCTLA-4-Fc, hCD2-Fc) or additional B7 family members (hB7.1-Fc, hB7.2-Fc, hB7-DC-Fc, hB7-H1-Fc, hB7-H2-Fc, hB7-H3-Fc) was similarly measured following the aforementioned protocols for hB7-H4-Fc.

For analyzing B7-H4-EGFP expression in transiently transfected 293F cells, a plasmid encoding full-length hB7-H4 (UniProt Q7Z7D3-1, residues 1–282), which includes the extracellular, transmembrane, and cytoplasmic domains, fused to an EGFP tag was constructed. 293F cells were transiently transfected with the plasmid using PEI-MAX transfection reagent. After two days of transfection, 5 × 10^5^ WT 293F and B7-H4-EGFP-expressing 293F cells were resuspended with 100 µL of PBS. Expression was quantified by flow cytometry as previously described.

For analyzing the binding of B7-H4 to activate human T cells, primary human T cells were activated with anti-human CD3/CD28 beads (Absin, abs160019) and cultured in RPMI-1640 medium containing 10% FBS and 1000 IU/mL IL-2. The activated human T cells were first stained with an amino-reactive dead cell dye (Thermo Fisher Scientific, L34975), and then incubated with hB7-H4-Fc or isotype control (human CD147-Fc) for 30 min at 4 °C. Following two washes with 0.5% BSA/PBS, the cells were stained with Alexa Fluor 647 anti-mouse Fc antibody for 20 min at 4 °C. Flow cytometry data acquisition and analysis were performed as described earlier. The binding of mouse B7-H4 to naive OT-1 T cells harvested from OT-1 mouse splenocytes and OVA-activated OT-1 T cells (cultured with 2 µg/mL OVA for 3 days) was similarly conducted by flow cytometry analysis as mentioned above.

To analyze immune cell subsets in spleens of Gal-9 KO mice, the spleens were processed into single-cell suspensions. After centrifugation at 300 g for 5 min, the splenic cells from half of the suspension were treated with Red Blood Cell Lysis Buffer to lyse red blood cells. Following this, the splenocytes were then filtered through a 40 µm cell strainer, and stained by an amino-reactive dead cell dye (Thermo Fisher Scientific, L34975) for 30 min at 4 °C. Following two washes with 0.5% BSA/PBS, cells were incubated with the primary antibodies targeting mouse immune lineages for 15 min at 4 °C. The flow cytometry data were acquired and analyzed as described above. Proportions of immune subsets within each parent gate were statistically analyzed using GraphPad Prism (v9.2.0).

### Animal studies

C57BL/6J mice (CD45.1^+^, CD45.2^-^) were obtained from Jackson Laboratory. BALB/c Nude mice, CB-17 SCID mice, and wild-type (WT) mice (C57BL/6J background) were obtained from Charles River. OT-1 mice were provided by Dr. Yulu Li at NIBS. Gal-9 KO mice, B7-H4 KO mice, and B7-H4/Gal-9 DKO mice were generated on a C57BL/6J background at the Transgenic Animal Center of NIBS. All mice were maintained under specific-pathogen-free conditions in the Animal Facility of NIBS. The animal experiments were conducted in accordance with the approved protocols of the Institutional Animal Care and Use Committee of NIBS (NIBS2022M0038).

For establishment of the adoptive transfer mouse model, naive OT-1 T cells (CD45.1^-^, CD45.2^+^) were isolated from OT-1 mouse splenocytes using Red Blood Cell Lysis Buffer. Cells were activated with 2 µg/mL OVA peptide for 3 days. Then OVA-activated OT-1 T cells (6.7 × 10^6^) were injected into the peritoneal cavity of each C57BL/6J mouse (CD45.1^+^, CD45.2^-^). On the next day (day 0) and day 7, 7 × 10^7^ EG7 tumor cells isolated from one EG7 tumor-bearing C57BL/6J mouse using Tumor Dissociation Kit (Miltenyi, 130-096-730), were injected into the peritoneal cavity of the C57BL/6J mouse (CD45.1^+^, CD45.2^-^). On day 1, host CD45.1^+^ cells, OT-1 T cells, or EG7 tumor cells within the peritoneal cavity of total two adoptive-transfer-model mice were harvested. The peritoneal immune cells were lysed with 1 mL of RIPA buffer (containing 1 mM proteinase PMSF) per up to 5 × 10^6^ cells for 45 min at 4 °C. Lysates were centrifuged at 12,000 g for 10 min. Aliquots of the supernatant were flash-frozen in liquid nitrogen and stored at −80 °C for use in the next step of the coprecipitation assay.

Gal-9 KO mice were obtained using cryopreserved sperm from Gal-9 KO mice (GemPharmatech) and *in vitro* fertilization was performed by Transgenic Animal Center at NIBS. Offspring were genotyped to confirm the Gal-9 KO genotype. The primers used for genotype PCR were as follows: F1 5’-TAGACTCCTACGTCCTGAGCATCCT-3’, R1 5’-ATCCAGATCAGGCAGCTCCTAAC-3’, F2 5’-CTTGTGTTTGCTTGCTTCATGC-3’, R2 5’-CTAGGACTTGCTTGTTAGGCAAGC-3’.

For generation of B7-H4 KO mice, Exon3 and Exon4, which encode the mouse B7-H4 IgV and IgC ectodomains, were selected as the KO region. The gRNAs were designed and synthesized by GENEWIZ. Microinjection of the gRNAs and CRISPR/Cas9 vector into mouse fertilized eggs was performed by Transgenic Animal Center at NIBS. Genotype primers were designed based on the different genomic sequences between WT mice and B7-H4 KO mice. Genotype PCR was conducted according to the manual of High-fidelity DNA polymerase KOD-Plus (TOYOBO) to confirm the B7-H4 KO genotype. The primers used for genotype PCR were as follows: F3 5’-GAGTTCCTTCATCATTCCAAGAAAGACAAAG-3’, R3 5’-TTGGTACCTACAGCA GATCTGTGCAC-3’, F4 5’-GAGTTCCTTCATCATTCCAAGAAAGACAAAG-3’, R4 5’-GGTATCTGATATGTAACCCCTGGAAAGAATTG-3’.

For generation of B7-H4/Gal-9 DKO mice, B7-H4 KO female mice served as the strain background. Cryopreserved sperm from Gal-9 KO male mice was used for *in vitro* fertilization. The resulting offspring were genotyped to confirm the B7-H4/Gal-9 DKO genotype using the genotype primers previously described for B7-H4 KO mice and Gal-9 KO mice.

For syngeneic mouse tumor models, including MC38 and MC38-mB7-H4 tumor models, male mice aged 10–12 weeks (BALB/c Nude, CB-17 SCID, C57BL/6J WT, B7-H4 KO, Gal-9 KO, or B7-H4/Gal-9 DKO) were inoculated subcutaneously with 5 × 10^5^ tumor cells in the right lower flank (day 0). Tumor dimensions were measured every 4 days using calipers. Tumor volume was determined according to the following formula based on caliper measurements: tumor volume = 0.5 × length × width^2^. In accordance with Ethical Approval for Research Involving Animals at NIBS, the experimental observation was terminated when mouse tumor volume approached 2000 mm^3^, typically occurring within 20–28 days.

### Humane endpoints and animal welfare

All animal experiments adhered to the guidelines of the Animal Welfare and Ethics Committee of NIBS. Animal care and experimental procedures were performed in strict compliance with the ethical approval and the animal welfare requirements specified in GB/T 35823−2018, ensuring no unintended survival disturbances unrelated to the experimental protocol. All research staff are qualified through national laboratory animal practitioner certification and have completed specialized in-house courses on animal care and experimental procedures at NIBS. They are proficient in relevant techniques and can perform animal experiments independently.

The predefined humane endpoints used for this study are as follows: (1) impaired mobility due to ascites or tumor burden; (2) total tumor volume approaching 2000 mm^3^ (prompt termination of the experiment); (3) loss of more than 20% body weight from baseline in mice resulting from model establishment and no significant recovery within 72 hours; (4) any condition that interferes with ability to eat/drink/ambulate; (5) excessive or prolonged hypothermia or hyperthermia; (6) anemia (pallor), jaundice; (7) skeletal fractures; (8) uncontrollable seizures, paralysis, severe ataxia (incoordination); (9) cyanosis, gasping, labored breathing; (10) severe, unresponsive diarrhea/vomiting, obstruction. Once animals met the endpoint criteria or the experimental protocol was completed, the experiment was terminated immediately, and euthanasia was performed typically within 30 minutes. All animals in this study were humanely euthanized upon reaching predefined humane endpoint criteria. No unanticipated or spontaneous deaths occurred prior to euthanasia.

The detailed methods of animal sacrifice, anesthesia and/or analgesia, and efforts to alleviate suffering are provided as follows. (1) Methods of sacrifice: mice were euthanized in an individually ventilated cage (IVC) using carbon dioxide (CO_2_) at a flow rate of 30–70% of the IVC volume per minute (equivalent to 3000–4000 mL/min). CO_2_ delivery was maintained for 3–6 minutes and then discontinued, after which the mice were retained in the IVC for an additional 2 minutes. Death was confirmed by assessing the absence of movement, cessation of breathing, and pupil dilation. (2) Method of anesthesia and/or analgesia: this study did not involve any survival surgical procedures, live surgery, or interventions expected to cause sustained pain beyond momentary discomfort (e.g., routine injections, tumor measurements). Therefore, the use of perioperative anesthesia or postoperative analgesia was not required by the experimental protocol. (3) Efforts to alleviate suffering: mice were housed in individually ventilated cages with ad libitum access to food and water. The housing environment was maintained at a temperature of 20–24 °C, relative humidity of 40–60%, and a 12-hour light/dark cycle. Animal health and behavior were monitored daily by the specialized animal caretakers of the Laboratory Animal Center of NIBS. For animals showing early signs of morbidity or those in high-risk groups, assessments and necessary interventions were performed within the same day by research staff. Main observation targets for the experiments were typically measured every 4 days.

Key welfare measures included: (1) separate housing of mice by gender, with timely cage separation if fighting occurred; (2) gentle handling to minimize stress; (3) utilization of alternatives and reduction of animal numbers whenever possible (animal studies were only designed if scientifically justified and necessary); (4) determination of sample size based on minimum statistical requirements to maximize experimental accuracy and animal utilization (the control group sample size could be smaller than that of the model group); (5) proficient performance of experimental procedures to minimize animal distress; (6) continuous monitoring of animal conditions, with prompt experimental termination if necessary; (7) use of appropriate euthanasia methods to alleviate suffering.

### Statistical analysis

Statistical comparisons among three or more groups were determined using one-way ANOVA and Tukey’s multiple comparisons test, while two-group comparisons were determined by unpaired Student’s t-test. All analyses were performed using GraphPad Prism (v9.2.0). Significance levels were defined as follows: *P* > 0.05 indicates no significant (ns) effect on the group means, and these *P* values signify that the mean of one group is significantly different from another, including * *P* < 0.05, ** *P* < 0.01, *** *P* < 0.001, **** *P* < 0.0001.

## Results

### Identification of Galectin-9 (Gal-9) as a B7-H4-binding protein

To elucidate the molecular mechanism underlying B7-H4’s negative immune-regulatory function, we first aimed to identify its potential binding partner(s). Preliminary evidence suggests that B7-H4 interacts with molecules expressed on activated—but not resting—T cells [15]. However, using recombinant human B7-H4 protein (hB7-H4-Fc, consisting of the human B7-H4 ectodomain, including both the IgV and IgC domains, fused to the mouse IgG2a Fc region), we found that the B7-H4 did not bind to activated primary human T cells (S1A Fig). Similarly, using recombinant mouse B7-H4 protein (mB7-H4-Fc, consisting of the mB7-H4 ectodomain, including both the IgV and IgC domains, fused to the human IgG1 Fc region), we found that mB7-H4 did not bind to naïve or antigen (OVA)-activated OT-1 T cells (S1B Fig).

**Fig 1:**
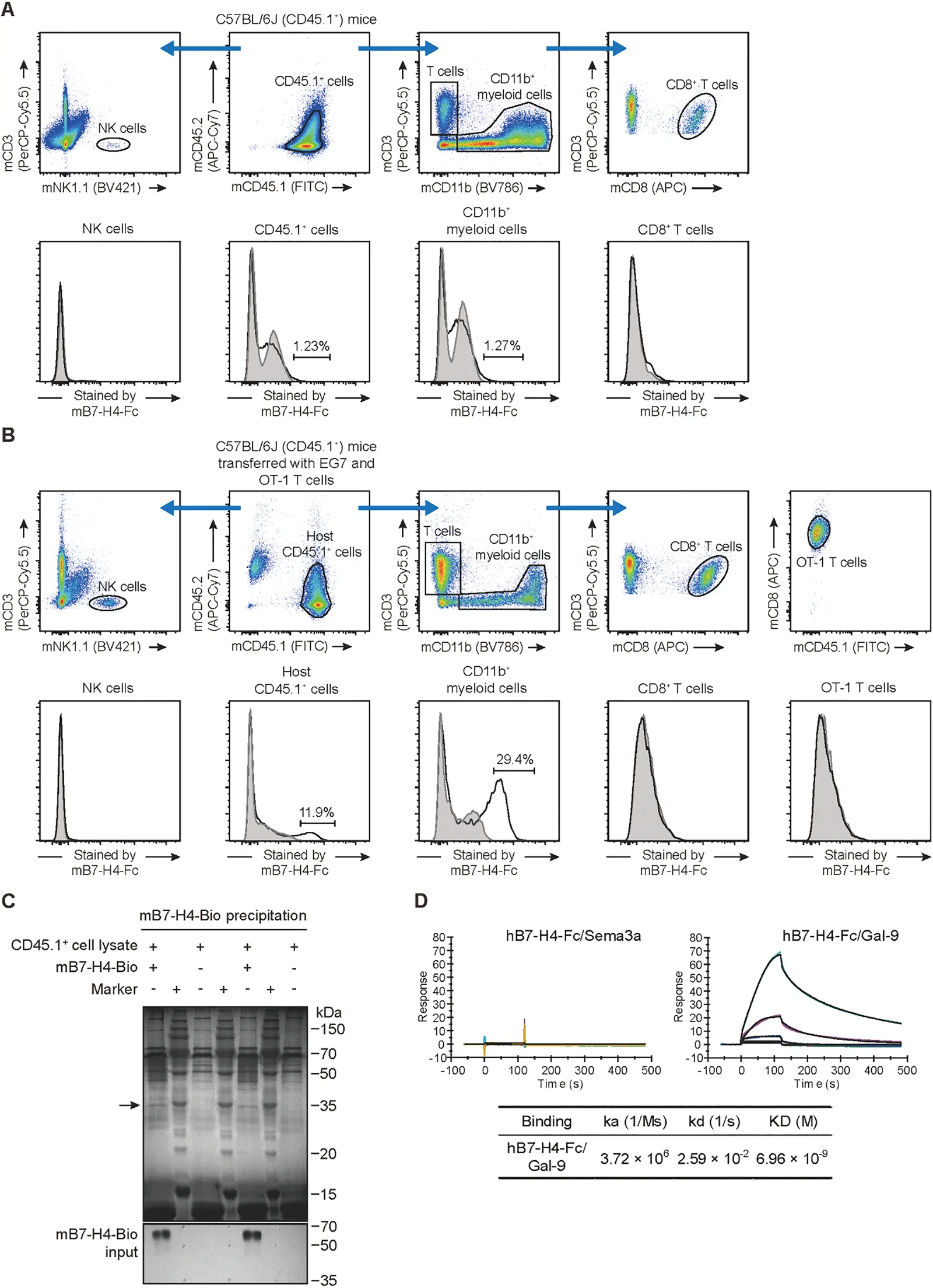
Identification of B7-H4’s binding partner, Gal-9. (**A-B**) Flow cytometry analysis of mB7-H4-Fc binding to peritoneal immune cells isolated from WT C57BL/6J (CD45.1^+^, CD45.2^-^) mice (A) and from C57BL/6J (CD45.1^+^, CD45.2^-^) mice one day after adoptive transferred with EG7 tumor cells and OT-1 T cells (B). Two-dimensional plots show the gating strategy to identify each immune cell lineage within the peritoneal immune cell population (top panels), and the blue arrows represent the sequence of gating. Histogram overlays show mB7-H4-Fc binding (black line) and isotype control binding (shaded gray) to each immune cell lineage (bottom panels). Fc-tagged proteins were tested at 10 µg/mL. Results from the same batch of adoptive-transfer animal models on day 7 are shown in S1C-S1D Figs. **(C)** Immunoprecipitation (IP) of peritoneal CD45.1 ⁺ cell lysates using biotinylated mB7-H4 (mB7-H4-Bio). Streptavidin beads captured mB7-H4-Bio-precipitated proteins were separated by SDS-PAGE and visualized by silver staining. The arrow indicates a specific ~35 kDa band precipitated by mB7-H4-Bio. The input mB7-H4-Bio protein was confirmed by western blotting with HRP-Streptavidin. Lanes 1 and 3, and lanes 5 and 7 represent two independent replicate pull-down experiments; lanes 2, 4 and 6 show protein markers. **(D)** SPR binding kinetics between hB7-H4-Fc and Gal-9. The Sema3a showed no binding to hB7-H4-Fc, while Gal-9 exhibited specific binding. The binding kinetic parameters (ka, kd, KD) are shown on the bottom panel. The binding between hB7-H4-Fc and Gal-9 was examined by SPR kinetic analysis in two independent experiments.

Since B7-H4 has been shown to be expressed on mouse peritoneal macrophages [3], we hypothesized that a putative mB7-H4 receptor may exist on immune cells within the mouse peritoneal cavity contributing to its immune-regulating function. To test this hypothesis, we first assessed the binding of mB7-H4-Fc to peritoneal immune cells from C57BL/6J mice (CD45.1^+^, CD45.2^-^) using flow cytometry. Only 1.23% of the CD45.1^+^ peritoneal immune cells were positive for mB7-H4-Fc binding (Fig 1A). In contrast, when analyzing peritoneal immune cells isolated from the same strain of mice one day after the adoptive transfer of EG7 tumor cells and OVA-specific OT-1 T cells (CD45.1^-^, CD45.2^+^), we observed that 11.9% of host CD45.1^+^ peritoneal immune cells especially 29.4% of host CD11b^+^ myeloid cells were positive for mB7-H4-Fc binding (Fig 1B). However, host NK cells, host CD8^+^ T cells, and the CD45.1^-^ OT-1 T cells within the peritoneal immune cells were found negative for mB7-H4-Fc binding (Fig 1B). After seven days of the adoptive transfer, 2.42% of host CD45.1^+^ peritoneal immune cells, 5.81% of host CD11b^+^ myeloid cells, and 2.02% of the CD45.1^-^ EG7 tumor cells showed mB7-H4-Fc positive binding (S1C-S1D Figs). These findings suggest that a putative mB7-H4 binding protein may be expressed or upregulated on the peritoneal immune cells in mice after the peritoneal adoptive transfer with EG7 tumor cells and OT-1 T cells.

To identify candidate binding proteins for mB7-H4, we next employed an immunoprecipitation (IP) coupled with mass spectrometry (MS) method. A biotinylated mB7-H4 recombinant protein (mB7-H4-Bio, see Methods) and M-280 Streptavidin magnetic beads were used to precipitate mB7-H4-Bio-binding proteins from the cell lysates of CD45.1^+^ peritoneal immune cells from the adoptive-transfer-model mice described above. Cell lysates from EG7 and 4T1 (a mouse breast cancer cell line) tumor cells were used as controls. The precipitated proteins were separated by SDS-PAGE electrophoresis and visualized by silver staining. A protein band with an apparent molecular weight of approximately 35 kDa was observed only when both mB7-H4-Bio and CD45.1^+^ peritoneal immune cell lysates were present; whereas this band was not observed when each component was tested alone (Fig 1C). Under other conditions, including those using both mB7-H4-Bio and 4T1 or EG7 cell lysates, mB7-H4-Bio did not preferentially precipitate any protein band that was absent in the corresponding no-mB7-H4-Bio control lane (S1E Fig). Collectively, the ~ 35 kDa band from CD45.1^+^ peritoneal immune cells was preferentially enriched by the bait protein mB7-H4-Bio. Next, this ~35 kDa band was excised from the gel, digested with trypsin, and subjected to MS analysis using LTQ-Orbitrap Velos (Thermo Fisher Scientific). The MS analysis identified mouse Galectin-9 (mGal-9) exhibited the highest abundance among all candidate binding proteins, with seven distinct mGal-9-matching peptides covering 26% of its protein sequence (S1F Fig). Notably, the theoretical molecular weight of mGal-9 (322 amino acids, 36.5 kDa), closely corresponds to the observed ~35 kDa band of interest. These results indicate that mGal-9 is a potential binding protein for mB7-H4.

To investigate whether the binding between mB7-H4 and mGal-9 is conserved in humans, we examined the binding of human B7-H4 to human Gal-9. Using surface plasmon resonance (SPR) (Biacore T200 system, GE Healthcare), we tested hB7-H4-Fc binding to a purified human Gal-9 protein (R&D systems). The result showed that hB7-H4-Fc specifically bound to the Gal-9 protein, but not to the purified human Sema3a protein (also served as a negative control; Fig 1D). These findings demonstrate the binding of B7-H4 and Gal-9 is conserved across species in both mice and humans.

### Binding of B7-H4 to Gal-9 is primarily glycan-mediated, involving B7-H4 ectodomain’s N-linked glycan, Gal-9’s N-CRD, and the R65 residue within the N-CRD

**Fig 2:**
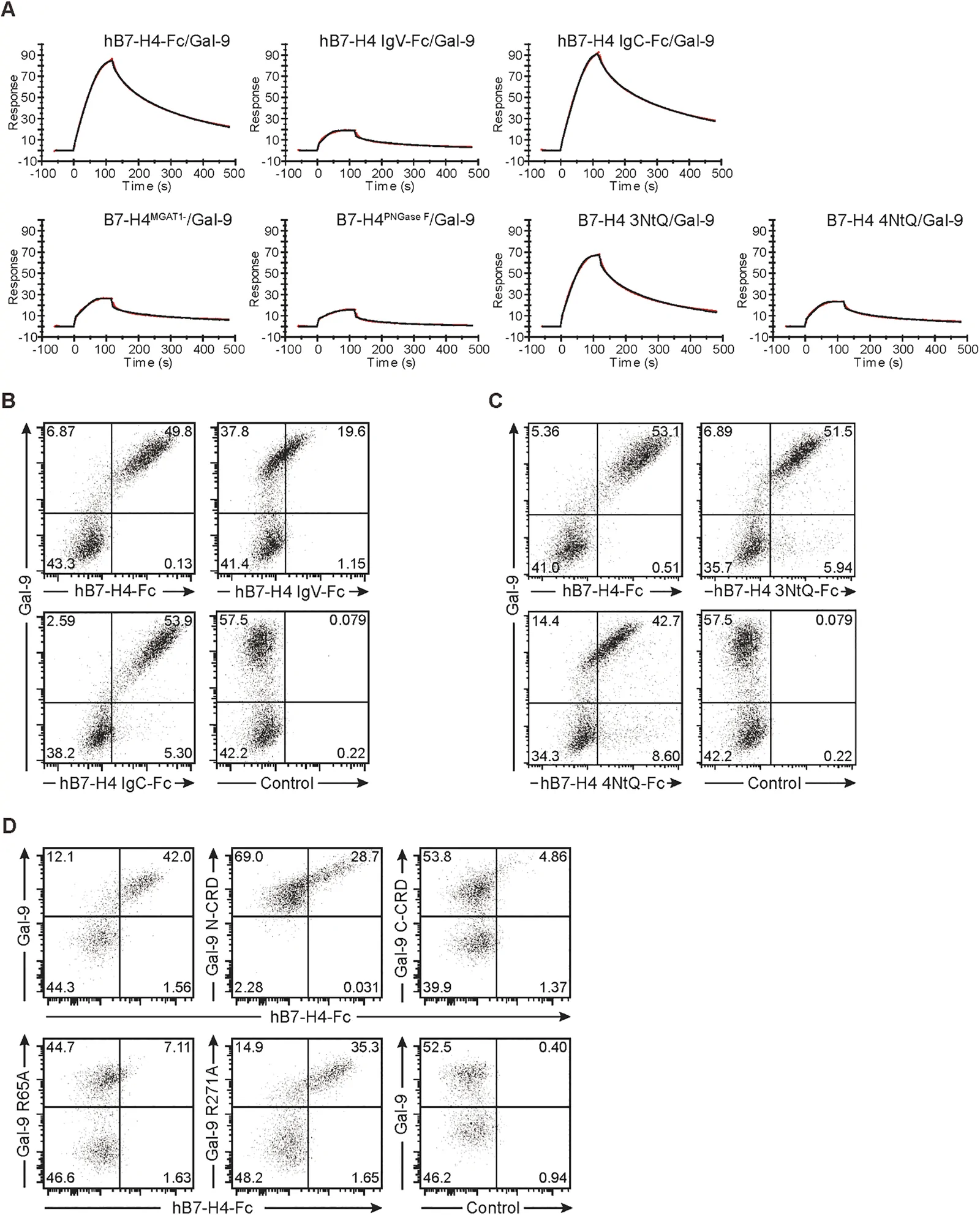
The R65 residue in the Gal-9 N-CRD mediates binding to glycosylated B7-H4 IgC domain. **(A)** The IgC domain and its glycosylation of B7-H4 are essential for its binding to Gal-9 protein. SPR binding kinetics are shown for hB7-H4-Fc and its various variants, including truncation variants (hB7-H4 IgV-Fc and hB7-H4 IgC-Fc), de-glycosylated forms (B7-H4^MGAT1-^ and B7-H4^PNGase F^), and mutants (B7-H4 3NtQ and B7-H4 4NtQ), in their binding to Gal-9 protein. Owing to incompatibility between PNGase F and its buffer with flow cytometry analysis, the two de-glycosylated proteins B7-H4^MGAT1-^ or B7-H4^PNGase F^ were examined by SPR analysis in two independent experiments. **(B-C)** The IgC domain of B7-H4 and its glycosylation are essential for the binding to Gal-9-expressing cells. hB7-H4-Fc and its truncation variants were analyzed for binding to Gal-9-EGFP-expressing 293T cells by flow cytometry (B). Likewise, hB7-H4-Fc and its glycosylation-deficient mutants were examined by flow cytometry (C). Using these two methodologically independent experimental approaches, binding of the same batch of B7-H4-Fc, B7-H4 IgV-Fc, B7-H4 IgC-Fc, B7-H4 3NtQ, and B7-H4 4NtQ to Gal-9 was evaluated by both SPR and flow cytometry, as depicted in panels A-C. **(D)** The R65 residue in the N-CRD of Gal-9 is required for its interaction with B7-H4. hB7-H4-Fc was examined for binding to Gal-9-EGFP-expressing 293T cells, Gal-9 truncation variants (Gal-9 N-CRD and Gal-9 C-CRD), or Gal-9 single-site mutants (Gal-9 R65A and Gal-9 R271A)-EGFP-expressing 293T cells by flow cytometry. Flow cytometry analysis of hB7-H4-Fc’s binding to Gal-9 or Gal-9 R65A mutant was independently replicated twice as shown in panel D and Fig 4B.

To further characterize the binding between B7-H4 and Gal-9, we investigated which Ig-like ectodomain of human B7-H4 mediates binding to human Gal-9. Two human B7-H4 ectodomain truncation variant proteins, the IgV-only variant (hB7-H4 IgV-Fc) and the IgC-only variant (hB7-H4 IgC-Fc), were produced similarly as the aforementioned hB7-H4-Fc (containing both IgV and IgC domains). SPR analysis revealed that hB7-H4 IgC-Fc bound to Gal-9 protein at levels comparable to hB7-H4-Fc, whereas the hB7-H4 IgV-Fc exhibited a markedly reduced binding (Fig 2A). Consistently, flow cytometry confirmed that hB7-H4 IgV-Fc showed reduced binding to 293T cells transiently expressing human Gal-9 (293T-Gal-9 cells), compared to both hB7-H4-Fc and hB7-H4 IgC-Fc (Fig 2B). Taken together, these data suggest that the IgC domain of B7-H4 is essential for its binding to Gal-9.

Given that Gal-9 is a lectin with carbohydrate recognition capabilities [23] and B7-H4 is a glycoprotein with seven potential N-linked glycosylation sites located across its IgV and IgC domains [1,5] (S2A Fig), the binding between B7-H4 and Gal-9 likely occurs in a glycan-mediated manner. It has also been reported that B7-H4’s glycosylation plays a crucial role in its stability and function [29]. To assess the role of B7-H4’s glycosylation in its binding with Gal-9, hB7-H4-Fc protein were de-glycosylated either by expressing it in *MGAT1* (encoding N-acetylglucosaminyltransferase I) gene KO 293F cells, which lack the ability to synthesize complex N-linked glycans [30], and naming it B7-H4^MGAT1-^, or by treating purified hB7-H4-Fc with PNGase F to remove N-linked glycans (B7-H4^PNGase F^). Both the de-glycosylated proteins, B7-H4^MGAT1-^ and B7-H4^PNGase F^, exhibited reduced binding to human Gal-9 protein compared to hB7-H4-Fc protein with glycosylation (Fig 2A), indicating the critical role of B7-H4’s glycosylation in mediating its binding with Gal-9.

To further investigate the specific glycosylation site involved, we generated B7-H4 mutants by substituting asparagine with glutamine at the predicted N-linked glycosylation sites using hB7-H4-Fc protein as the backbone to produce seven single-site mutants: N112Q in the IgV domain, and six in the IgC domain (N160Q, N190Q, N196Q, N205Q, N216Q, and N220Q), and two multi-site mutants: B7-H4 3NtQ (N205Q, N216Q, and N220Q) and B7-H4 4NtQ (N112Q, N160Q, N190Q, and N196Q) (S2A-S2B Figs). SPR analysis showed that all seven single-site mutants (S2C Fig) and the multi-site mutant B7-H4 3NtQ exhibited minimal changes in Gal-9 binding compared to WT hB7-H4-Fc; whereas the B7-H4 4NtQ mutant showed weaker Gal-9 binding than WT hB7-H4-Fc (Fig 2A). Consistently, flow cytometry confirmed that the B7-H4 4NtQ mutant showed reduced binding to 293T-Gal-9 cells, compared to WT hB7-H4-Fc (Fig 2C). These results demonstrate that glycosylation at N160, N190, and N196 in the IgC domain of human B7-H4 ectodomain are critical for its binding to human Gal-9, consistent with the observation that the IgC domain is essential for this interaction.

Human Gal-9 consists of two CRDs, N-terminal CRD (N-CRD) and C-terminal CRD (C-CRD). To identify which CRD of Gal-9 is involved in binding with human B7-H4, we transiently overexpressed each of the CRD domains of human Gal-9 (UniProt O00182-1) in 293T cells and generated the N-CRD-only variant (293T-Gal-9 N-CRD) cells and the C-CRD-only variant (293T-Gal-9 C-CRD) cells, similar to that for generating WT 293T-Gal-9 cells described above. The binding of hB7-H4-Fc to these cells was assessed using flow cytometry. We found that hB7-H4-Fc bound to 293T-Gal-9 N-CRD cells at slightly lower levels compared to WT 293T-Gal-9 cells, whereas its binding to 293T-Gal-9 C-CRD cells was markedly weaker (Fig 2D). To further investigate the specific residues involved in the binding, two Gal-9’s loss-of-function single-site mutants [24], R65A in the N-CRD and R271A in the C-CRD (corresponding to R239A in the short isoform of Gal-9 (UniProt O00182-2)) were similarly expressed in 293T cells. Flow cytometry analysis revealed that hB7-H4-Fc bound to 293T-Gal-9 R271A cells at levels comparable to 293T-Gal-9 cells, whereas its binding to 293T-Gal-9 R65A cells was markedly reduced (Fig 2D). Taken together, these results demonstrate that the binding between human B7-H4 and human Gal-9 is mediated by the glycosylated B7-H4 ectodomain, particularly the IgC domain, and the N-CRD domain of Gal-9, with the R65 residue in the N-CRD serving as a critical determinant of this binding.

### B7-H4 binding to Gal-9 attenuates Gal-9-induced T cell death

Gal-9 is recognized as an immune-regulator with diverse but not yet fully understood mechanisms. It plays a dual role in immunoregulation, capable of either stimulating or suppressing the immune response [31]. Its activity on T cells is believed to depend on whether it is localized intracellularly or extracellularly [16,32]. While Gal-9 is predominantly localized within intracellular compartments in CD4^+^ and CD8^+^ T cells, it translocates to the T cell membrane upon activation. Notably, exogenous soluble Gal-9 is recognized for its role in inducing T cell apoptosis [23,33,34], a process initially believed to be mediated by TIM-3 expressed on T cells [23]. However, subsequent studies revealed that TIM-3 is not the sole mediator of this mechanism [35,36]. This prompted us to first investigate whether B7-H4 participates in Gal-9-induced T cell apoptosis.

We first sought to confirm that Gal-9 induces T cell death in primary human T cells. To this end, human peripheral blood mononuclear cells (PBMCs) were incubated with Gal-9 for 0.5 hours (h), and the cell viability was analyzed using flow cytometry. Compared with untreated controls, Gal-9 treatment significantly reduced immune cell viability: total PBMC viability decreased from 79.8% to 47.4%, CD3^+^ T-cell viability from 87.2% to 27.8%, and CD19^+^ B-cell viability from 91.1% to 31.7%, and CD14^+^ myeloid-cell viability from 60.8% to 30.6% (S3A-S3E Figs). Directly treating primary T cells isolated from PBMCs with Gal-9 for either 0.5 h or 18 h also significantly reduced the T cell viability (S3F-S3G Figs). Collectively, these results indicate that Gal-9 induces immune cell death.

**Fig 3:**
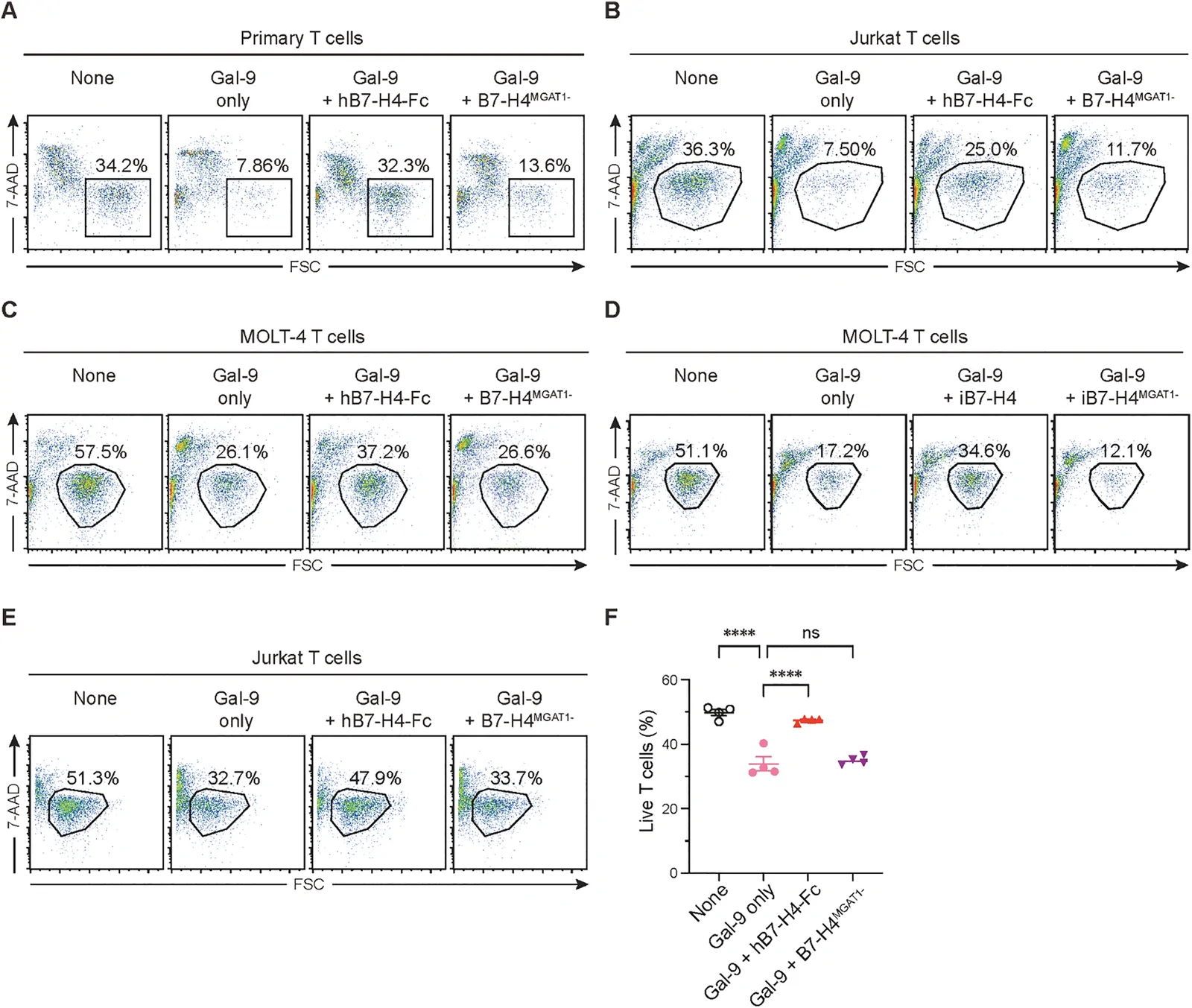
B7-H4 suppresses Gal-9-induced T cell death, and this suppression depends on the glycosylation of B7-H4. **(A-C)** Flow cytometry analysis of live cells (7-AAD negative) within primary human T cells (A), Jurkat T cells (B), and MOLT-4 T cells (C) after treatment with 8 µg/mL Gal-9 for 30 min in the presence or absence of 25 µg/mL glycosylated hB7-H4-Fc, or de-glycosylated hB7-H4-Fc (B7-H4^MGAT1-^). **(D)** Flow cytometry analysis of the proportion of live T cells (7-AAD negative) within the parent gate of MOLT-4 T cells treated with 6 µg/mL Gal-9 for 30 min in the presence or absence of Protein A-immobilized glycosylated hB7-H4-Fc (iB7-H4) or de-glycosylated hB7-H4-Fc (iB7-H4^MGAT1-^). (**E-F**) Flow cytometry analysis of T-cell viability in Jurkat T cells treated with Gal-9 and hB7-H4-Fc. Cells were treated with 6 µg/mL Gal-9 and/or 5 µg/mL hB7-H4-Fc or B7-H4^MGAT1-^. Representative dot plots from one independent experiment are shown in panel E. Statistical analysis of the Jurkat T-cell viability across all treatment groups is shown in panel F. Four independent replicates were performed for each condition. One-way ANOVA was used to compare T-cell viability among the four treatment conditions. Data are presented as mean  ±  s.e.m.

Having confirmed that Gal-9 induces T cell death, we next tested whether B7-H4 inhibits this effect in primary T cells, Jurkat T cells (a human T lymphoblastoid cell line), and MOLT-4 T cells (a human T lymphoblast cell line). Each of these cell types was incubated with Gal-9 for 0.5 h with or without hB7-H4-Fc or de-glycosylated B7-H4^MGAT1-^, followed by cell viability analysis using flow cytometry. We found that hB7-H4-Fc markedly reduced Gal-9-induced T cell death, whereas B7-H4^MGAT1-^ had much less or minimal effect in all the three cell types examined, including primary T cells (Fig 3A), Jurkat T cells (Fig 3B), and MOLT-4 T cells (Fig 3C). Another form of B7-H4, iB7-H4 (immobilized hB7-H4-Fc on Protein A beads), similarly reduced Gal-9-induced T cell death and de-glycosylated iB7-H4^MGAT1-^ had no effect on Gal-9-induced T cell death in MOLT-4 T cells (Fig 3D). We confirmed that glycosylated hB7-H4-Fc significantly reduced Gal-9-induced T cell death in Jurkat T cells compared to those only treated with Gal-9 (Figs 3E-3F). Additionally, hB7-H4-Fc significantly reduced Gal-9-induced T cell death in MOLT-4 T cells (S4F-S4G Figs). Together, these results demonstrate that B7-H4 attenuates Gal-9-induced T cell death, and this process is glycosylation-dependent, consistent with its glycosylation-dependent binding to Gal-9.

Nonetheless, this finding appears contradictory to B7-H4’s known inhibitory immune-regulating role. Since we did not observe direct binding of B7-H4 to activated T cells (S1A-S1B Figs), we hypothesize that B7-H4’s inhibition of Gal-9-induced T cell death is likely indirect, mediated through competition with Gal-9’s binding partners on T cells. Identifying these other, as-yet-unknown Gal-9 binding partners on T cells may shed light on the potential complexity of B7-H4’s inhibitory immune-regulating mechanism.

### Gal-9 binds to several T cell surface immune receptors, including CD28, and B7-H4’s binding to Gal-9 interferes with Gal-9-induced CD28 activation

**Fig 4:**
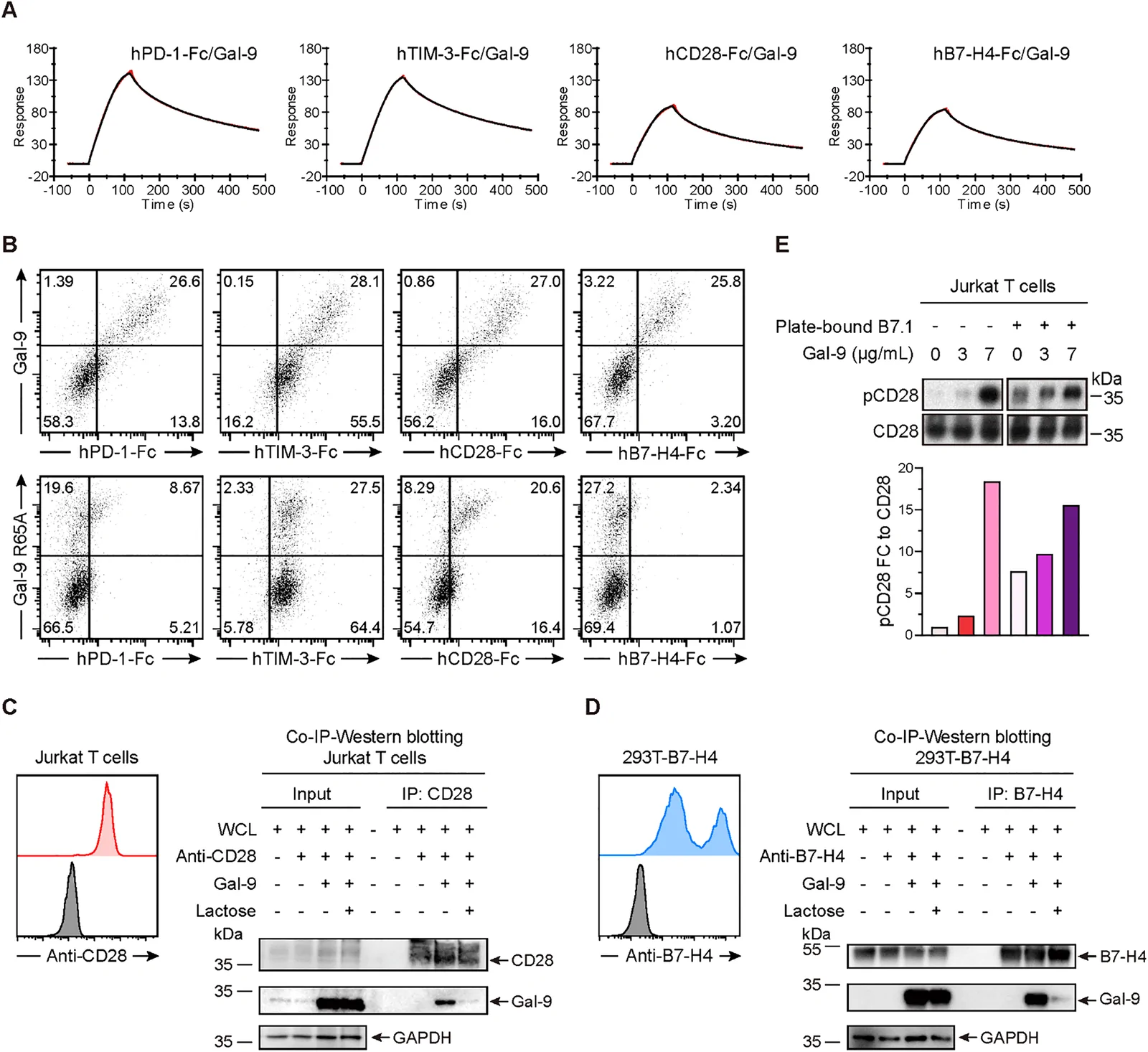
Gal-9 binds to CD28 and enhances the stimulation of pCD28 signaling activity. **(A)** SPR analysis of Gal-9 binding kinetics to PD-1, TIM-3, CD28, and B7-H4. Human PD-1, TIM-3, and CD28 were fused to the same mouse IgG2a Fc tags. 1.2 µg/mL Gal-9 was injected over a Protein A/G-coated Biacore chip to assess binding kinetics to each immobilized Fc-fusion protein. **(B)** Flow cytometry analysis of the binding between the four proteins (shown in Panel A) and 293T cells transiently overexpressing either WT Gal-9 or the Gal-9 R65A mutant. (**C**) Cell-surface binding of CD28 to Gal-9 analyzed by Co-IP-Western blotting. Cell-surface expression of CD28 on Jurkat T cells was examined by flow cytometry (left panel); CD28 expression was detected using an anti-CD28 antibody (red), with a cell-only control included (black). For the Co-IP-Western blotting analysis (right panel); 400 µL of whole cell lysates (WCL) from Jurkat T cells were incubated with or without 4 µg/mL Gal-9 and 200 mM lactose, followed by IP using an anti-CD28 antibody. Bound proteins were eluted and analyzed by Western blotting using a different anti-CD28 antibody from that used for IP, together with an anti-Gal-9 antibody, as indicated. (**D**) Cell-surface binding of B7-H4 to Gal-9 was analyzed by Co-IP-Western blotting. Cell-surface expression of B7-H4 on 293T cells transiently transfected with B7-H4 (293T-B7-H4) was examined by flow cytometry (left panel); B7-H4 expression was detected with an anti-B7-H4 antibody (blue), with a cell-only control included (black). For the Co-IP-Western blotting analysis (right panel), 400 µL of WCL from 293T-B7-H4 cells were incubated with or without 4 µg/mL Gal-9 and 200 mM lactose, followed by IP using an anti-B7-H4 antibody. Bound proteins were eluted and analyzed by Western blotting using a different anti-B7-H4 antibody from that used for IP, and an anti-Gal-9 antibody, as indicated. Anti-GAPDH antibody was used as an internal loading control for Western blotting of WCL samples in (C) and (D). **(E)** Gal-9-mediated stimulation of pCD28 signaling activity in Jurkat T cells. Jurkat T cells were treated with or without plate-bound B7.1, and Gal-9’s dose-dependent effects on pCD28 were analyzed by western blotting. Fold change (FC) in pCD28 band intensity relative to the CD28 loading control was quantified using ImageJ and GraphPad Prism.

To explore this, we first examined the binding of Gal-9—11 additional immune receptors primarily expressed on T cells (some are also expressed on NK cells or other immune cells), as well as its two known binding partners, TIM-3 and PD-1. These receptors were produced as recombinant proteins and tested for binding to Gal-9 protein using SPR. Additionally, they were also tested for binding to 293T-Gal-9 cells using flow cytometry. Of the 11 additional receptors tested, CD28, 2B4 (CD244 or SLAMF4), CD226, and SLAMF1 bound to Gal-9 at levels comparable to PD-1, TIM-3, and B7-H4 in SPR and/or flow cytometry assays (Figs 4A-4B and S4A-S4B Figs). By comparison, the remaining seven immune receptors showed weak or minimal binding to Gal-9 in SPR analysis (S4A Fig).

Comparative analysis revealed that 2B4, CD28, and SLAMF1, similar to TIM-3, bound to 293T-Gal-9 R65A cells at levels comparable to or only slightly reduced relative to 293T-Gal-9 cells. Conversely, CD226, like PD-1 and B7-H4, showed markedly reduced binding to 293T-Gal-9 R65A cells compared to 293T-Gal-9 cells (Fig 4B and S4B Fig). These results indicate that Gal-9 R65 residue in N-CRD differentially mediates interactions between Gal-9 and its binding partners. This is consistent with established findings that the Gal-9 R65 residue is dispensable for Gal-9’s binding to TIM-3 but plays a role in its binding to PD-1 [24].

Conversely, we examined whether cell surface-expressed immune receptors can interact with Gal-9. A previous study reported the interactions of cell-surface PD-1 and TIM-3 with Gal-9 via Co-IP-Western blotting [24]. Using similar methods, we examined and confirmed the interactions of Gal-9 with cell-surface CD28 and B7-H4. Cell-surface CD28 on Jurkat T cells as well as cell-surface B7-H4 interacted with Gal-9 and such interactions were inhibited by lactose (Figs 4C-4D). These results suggest that cell-surface CD28 and B7-H4 also directly interact with Gal-9 in a glycosylation-dependent manner.

As CD28 is a pivotal co-stimulatory receptor that potentiates TCR signaling to drive T cell expansion, cytokine secretion, and survival [37], we next focused our efforts on analyzing the effect of Gal-9 on T cells through CD28 engagement and whether B7-H4’s binding to Gal-9 indirectly interferes with this effect. As phosphorylation of CD28 (UniProt P10747-1) at tyrosine 191 (pCD28) is essential for PI3K-AKT pathway activation and subsequent T cell proliferation [38,39], we assessed Gal-9’s effect on CD28 signaling. In Jurkat T cells, Gal-9 induced pCD28 similarly to the plate-bound B7.1, a canonical CD28 agonist. Furthermore, Gal-9 exhibited additive effects with B7.1 in inducing pCD28, as evidenced by the increased phosphorylation levels of CD28 in the presence of plate-bound B7.1 (Fig 4E).

**Fig 5:**
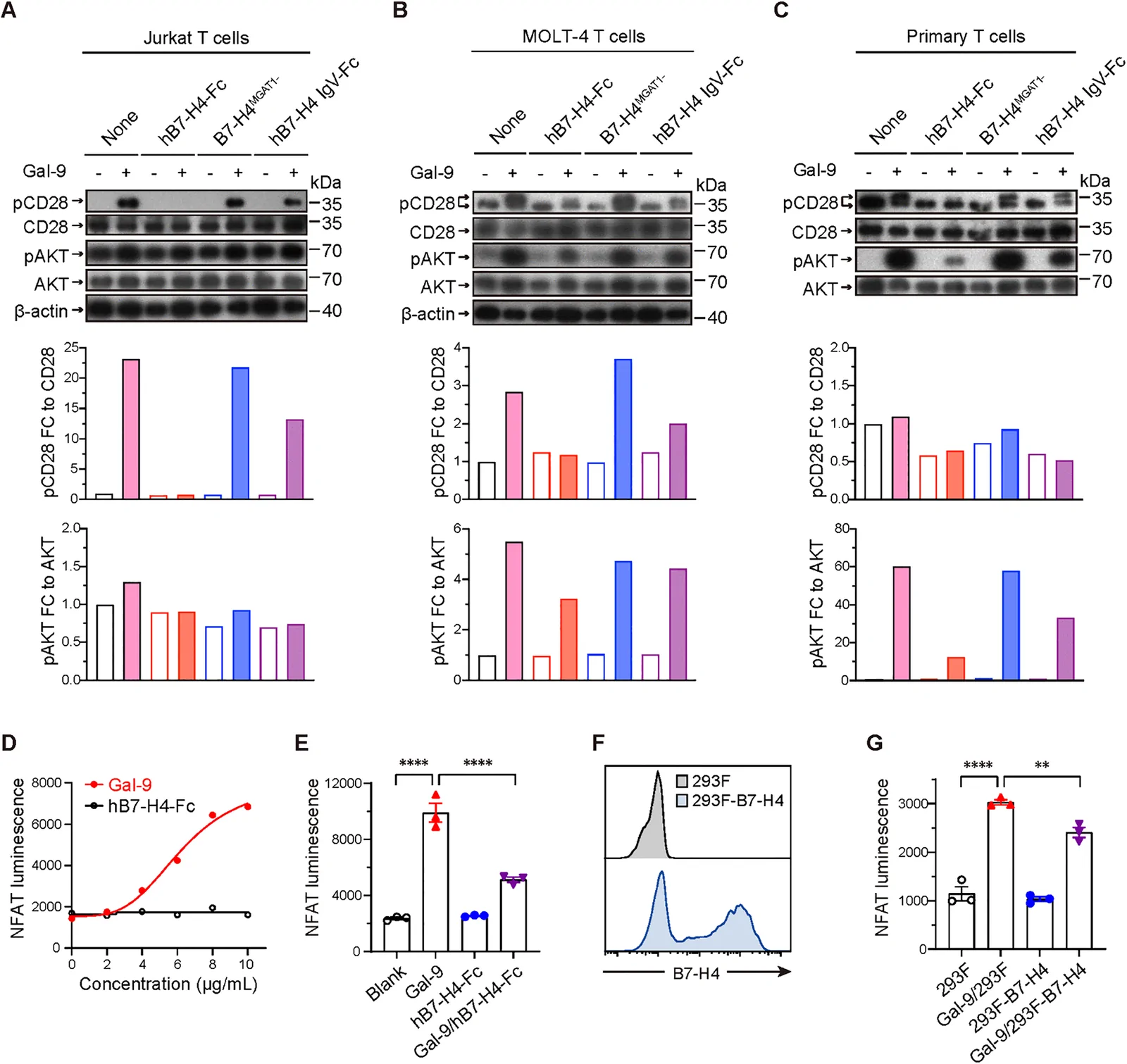
Glycosylated B7-H4 attenuates Gal-9-mediated CD28/AKT activation in human T cells. **(A-C)** B7-H4 modulates pCD28 and pAKT signaling in T cells in a glycosylation-dependent manner. Jurkat (A), MOLT-4 (B), and primary T cells (C) were treated with 8 µg/mL Gal-9 in the presence or absence of 25 µg/mL hB7-H4-Fc, hB7-H4 IgV-Fc, or de-glycosylated B7-H4^MGAT1-^. Western blotting was performed to detect pCD28 and pAKT signaling activity (top panels). Fold changes (FC) in pCD28 and pAKT band intensity relative to the corresponding CD28 and AKT loading controls were quantified using Image J and GraphPad Prism (middle and bottom panels, respectively). Gal-9-mediated stimulation of pCD28 and pAKT signaling, and its regulation by hB7-H4-Fc, were independently replicated in Jurkat (Panel A and Fig 4E), MOLT-4 T cells (Panel B and S4C-S4E Figs), and primary T cells (Panel C and S5C Fig) in two or three experiments. **(D)** Gal-9 stimulates NFAT signaling. Jurkat-NFAT-Luc reporter cells showed dose-dependent luciferase induction by Gal-9, but not hB7-H4-Fc. **(E)** B7-H4 attenuated Gal-9-induced CD28 signaling. Jurkat-NFAT-Luc reporter cells were treated with 3.3 µg/mL Gal-9, 25 µg/mL hB7-H4-Fc, or both. **(F)** Flow cytometry analysis of B7-H4 expression on 293F cells. B7-H4 expression on 293F cells (293F-B7-H4) was confirmed, the 293F-B7-H4 cells were used in panel G. **(G)** Cell-surface B7-H4 attenuated Gal-9-induced CD28 signaling. Jurkat-NFAT-Luc reporter cells were co-cultured with 293F or 293F-B7-H4 cells in the presence or absence of 3.3 µg/mL Gal-9. One-way ANOVA was used to statistically analyze the mean of NFAT-mediated luminescence signals among groups in Panels E and G. All data are presented as mean  ±  s.e.m.

Gal-9 also induced pCD28 signaling activity in MOLT-4 T cells, as well as in primary human T cells, consistent with the observations in Jurkat T cells (Figs 5A**-**5C). Further testing revealed that soluble hB7-H4-Fc inhibited this Gal-9-induced pCD28 in all three cell types, while the two Gal-9 binding-weakened variants, hB7-H4 IgV-Fc and the de-glycosylated B7-H4^MGAT1-^ showed either abolished or markedly reduced inhibition of pCD28 compared to hB7-H4-Fc. Additionally, Gal-9 induced phosphorylation of AKT (UniProt P31749-1) at serine 473 (pAKT) in both MOLT-4 and primary T cells (S4C-S4D Figs and Figs 5B-5C). The pAKT levels were significantly inhibited by hB7-H4-Fc treatment in MOLT-4 T cells compared to those only induced by Gal-9 (S4E Fig), whereas its two variants exhibited noticeably weaker inhibitory effects on pAKT in both MOLT and primary T cells, albeit to varying degrees (Figs 5B-5C).

Phosphorylated AKT activates downstream NFAT signaling via GSK3β inhibition, upregulating IL-2 expression and promoting T cell proliferation [37,40,41]. Using Jurkat-NFAT-Luc reporter cells (see Methods) where NFAT drives luciferase expression, we observed that Gal-9 induced NFAT-driven luciferase activity in a dose-dependent manner, while hB7-H4-Fc showed no such effect even at the highest concentration tested (10 µg/mL) (Fig 5D). Additionally, hB7-H4-Fc potently suppressed Gal-9-mediated NFAT activation (Fig 5E); similarly, 293F cells overexpressing cell-surface B7-H4 (293F-B7-H4, Fig 5F) also significantly inhibited Gal-9-mediated NFAT activation (Fig 5G). Collectively, these results demonstrate that Gal-9’s binding with cell-surface CD28 induces CD28 signaling activation (pCD28/pAKT/NFAT) in human T cells; B7-H4 inhibits this effect, regardless of whether B7-H4 is in its soluble or cell-surface form, likely through competitive binding to Gal-9, indirectly interfering with this signaling activation.

### B7-H4 is insufficient to alter Gal-9-mediated reduction of splenic CD4^+^ T cells but it remains immunoregulatory activities in mice

Given the similarity of B7-H4 to other members in the B7 family and their extensive glycosylation [10], we next examined whether the other six B7 family members that are not expressed on T cells could also bind to Gal-9 in a manner similar to B7-H4. Using SPR and flow cytometry, we found that soluble extracellular domains of four B7 member proteins—B7-H2, B7.1, B7-DC, and B7.2—bound Gal-9 with equal to or greater affinity than hB7-H4-Fc. In contrast, B7-H3 and B7-H1 exhibited weaker binding activity (S5A-S5B Figs). Distinct from hB7-H4-Fc’s effect on Gal-9-mediated stimulation of pAKT and induction of T cell death, hB7-H3-Fc and hB7-H1-Fc had a negligible effect on Gal-9’s activity in human primary T cells (S5C-S5D figs). These findings suggest that B7 family members, Gal-9, and other immune cell surface receptors may form a complex regulatory network that modulates T cell activity.

**Fig 6:**
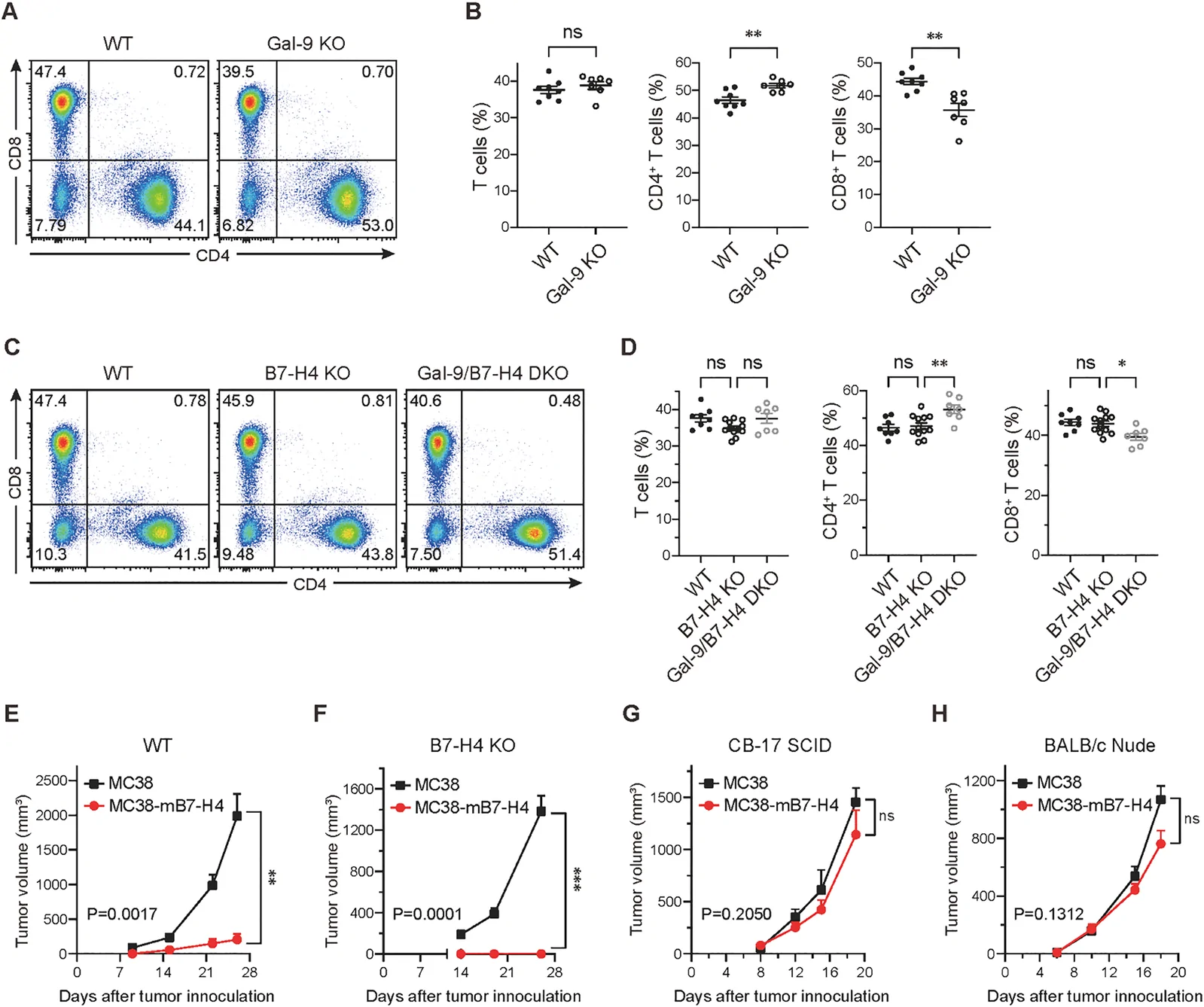
Gal-9 knockout increases the proportion of splenic CD4^+^ T cells in mice. **(A-B)** Flow cytometry analysis of splenic CD4^+^ and CD8^+^ T-cell proportions in WT and Gal-9 KO mice. Representative dot plots gated on live CD45^+^ cells from one mouse are shown in panel A. Summary data and statistical analysis of total T cells, CD4^+^ T cells, and CD8^+^ T cells are shown in panel B. n = 8 mice for the WT group; n = 7 mice for the Gal-9 KO group. (**C-D)** Flow cytometry analysis of splenic CD4^+^ and CD8^+^ T-cell proportions in WT, B7-H4 KO, and B7-H4/Gal-9 DKO mice. CD45^+^ dead-cell analysis is shown in S6F Fig. Panels C and D are arranged similarly to panels A and B, respectively. n = 8 mice for the WT group; n = 12 mice for the B7-H4 KO group; n = 7 mice for the B7-H4/Gal-9 DKO group. **(E-H)** Tumor growth comparison of MC38 and MC38-mB7-H4 tumors in WT C57BL/6J mice (E), B7-H4 KO mice (F), CB-17 SCID mice (G), and BALB/c Nude mice (H). In each mouse model, MC38 and MC38-mB7-H4 tumor cells were implanted bilaterally, with MC38 on the right flank and MC38-mB7-H4 on the left flank; the two tumor groups therefore shared the same mice (n = 4 mice per group). Cell-surface mB7-H4 expression on MC38 and MC38-mB7-H4 cells is shown in S7A Fig. Statistical significance was determined using an unpaired t-test for panel B, one-way ANOVA for panel D, and two-way ANOVA for panels E-H. Data are presented as mean ± s.e.m.

To further explore the physiological role of B7-H4 binding to Gal-9 *in vivo*, Gal-9 KO mice, B7-H4 KO mice, and B7-H4/Gal-9 DKO mice were generated (S6A-S6E Figs) and were phenotyped for splenic immune cell proportions. Immunophenotyping analysis of adult Gal-9 KO mice showed that mGal-9 deficiency was associated with a significant lower proportion of CD45^+^ dead cells among total splenocytes compared with WT mice (S6F Fig). A significant increase in the proportion of CD4^+^ T cells and a significant decrease in the proportion of CD8^+^ T cells in Gal-9 KO mice were observed compared to those in WT mice, whereas no significant difference in the proportions of total T cells was observed (Figs 6A-6B), as well as B cells, NK cells, or CD11b^+^ cells between Gal-9 KO and WT mice (S6G Fig). These results indicate that CD4^+^ and CD8^+^ T cells are the primary immune lineages affected by Gal-9 deficiency in mice. Considering that Gal-9 induces T-cell death *in vitro*, mGal-9 deficiency reduced the proportion of CD45^+^ dead cells, while CD4^+^ T cells were the only major immune-cell population significantly increased *in vivo*, we therefore focused our analysis on CD4^+^ T cells and hypothesize that Gal-9 results in the reduction of splenic CD4^+^ T cells during T cell development.

Further analysis of B7-H4/Gal-9 DKO mice revealed a significant increase in the proportion of splenic CD4^+^ T cells and a significant decrease in the proportion of splenic CD8^+^ T cells compared to B7-H4 KO mice (Figs 6C**-**6D), similar to what was observed in Gal-9 KO mice. However, B7-H4 loss alone in wild-type C57BL/6J mice possessing intact Gal-9 exhibited no significant changes in the decreased proportion of CD4^+^ T cells and increased proportion of CD8^+^ T cells driven by mGal-9 *in vivo* (Fig 6D). These results suggest that B7-H4 KO alone is insufficient to alter Gal-9’s activity in regulating splenic immune cell composition *in vivo*. This is consistent with the earlier observation that multiple B7 family members and immune receptors on T cells interact with Gal-9.

Additionally, we examined the role of host tissue-expressed B7-H4 and Gal-9 in tumor progression by assessing tumor growth in B7-H4 KO, Gal-9 KO, and B7-H4/Gal-9 DKO mice. Using the murine colon adenocarcinoma cell line MC38, which lacks cell-surface mB7-H4 expression (S7A Fig)—thereby precluding recognition of B7-H4 as a foreign antigen in B7-H4 KO or B7-H4/Gal-9 DKO mice—we found no difference in MC38 tumor growth among the three KO groups (S7B Fig). These results indicate that host-expressed B7-H4 and Gal-9 are not critical for tumor progression in these models. Intriguingly, contrary to previous reports suggesting tumor cell-expressed B7-H4 promotes tumor growth in WT immunocompetent mice [42], we found that mB7-H4-overexpressing MC38 cells (MC38-mB7-H4) (S7A Fig) exhibited significantly reduced tumor growth in WT mice (Fig 6E). This observation suggests that mB7-H4 overexpression on tumor cells does not promote tumor growth. Instead, it may activate the mouse immune response by interacting with unknown receptors or ligands, thereby enhancing antitumor activity. However, MC38-mB7-H4 cells failed to establish tumors in B7-H4 KO mice (Fig 6F), suggesting mouse B7-H4 is recognized as a foreign antigen in the B7-H4 KO mice. The MC38-mB7-H4 tumor cells exhibited similar growth patterns to MC38 in two immunodeficient mouse models, with no significant differences observed in tumor growth curves (Figs 6G**-**6H). Collectively, our findings indicate that B7-H4 is insufficient to alter the Gal-9-mediated reduction of splenic CD4^+^ T cells but may play a redundant role in immune regulation.

## Discussion

In this study, we identified Gal-9 as a previously unknown binding partner for B7-H4. Additionally, we uncovered several other previously unrecognized Gal-9 binding partners, including T cell surface receptors and multiple B7 family members. The molecular interaction between Gal-9 and B7-H4 is predominantly mediated by the glycan in the IgC domain of B7-H4 and the R65 residue in Gal-9’s N-CRD domain. Similarly, other binding partners also rely on their glycosylation for Gal-9 binding, while the R65 residue in Gal-9’s N-CRD domain is either dispensable or essential, depending on the binding partner. Moreover, both soluble and cell-surface B7-H4 counteract Gal-9-induced T cell death and Gal-9-induced CD28 activation *in vitro*. While Gal-9 KO in mice resulted in an increase in CD4^+^ T cells within the splenic immune cell composition, B7-H4 KO had no effect on splenic immune cell composition. The DKO of B7-H4 and Gal-9 showed no additional effect compared to the *in vivo* activity observed with Gal-9 KO alone. Furthermore, Gal-9 KO, B7-H4 KO, or DKO of both Gal-9 and B7-H4 did not alter anti-tumor immunity in MC38 tumor-bearing C57BL/6 mouse model. Our findings indicate that it is likely B7-H4, in conjunction with T cell surface immune receptors, interacts with Gal-9 as part of a complex regulatory network that finely modulates T cell activity through multiple and overlapping pathways.

The identification of B7-H4’s binding partner or functional receptor has been challenging due to the lack of B7-H4 binding target cells. It is worth noting that, in this study, we identified Gal-9 as a binding partner for B7-H4 using peritoneal immune cells isolated from mice adoptively transferred with OVA-expressing EG7 tumor cells and OVA-specific OT-1 T cells as the target cells bound by B7-H4 in the IP-MS method. It is apparent that Gal-9 is either induced to be expressed or markedly upregulated in peritoneal immune cells following the adoptive transfer of EG7 and OT-1 cells in mice, though the mechanism underlying this induction remains unknown. Notably, the peritoneal OT-1 cells themselves did not bind to B7-H4; instead, it was host peritoneal immune cells bound to B7-H4. We surmise that the immune response, not necessarily limited to the OVA-specific OT-1 T cell response, following adoptive transferring of both tumor cells and OT-1 cells, induced upregulation of Gal-9 expression in peritoneal host immune cells. Indeed, previous reports indicate that Gal-9’s expression can be induced by various factors, such as IFN-g, IL-1b, and TNFa [31].

Interestingly, B7-H4, a member of the B7 family and the immunoglobulin superfamily, has been proposed to be a glycosylphosphatidylinositol (GPI)-anchored cell surface membrane protein [3,43]. B7-H4 exhibits low to undetectable protein expression levels in most healthy tissues [5,10]. The ectopic expression of B7-H4 in various human cancers, including melanoma, esophageal squamous cell carcinoma, and gastric cancer, has been shown to correlate closely with tumor progression and patient survival outcomes [44–46]. Collectively, B7-H4 is widely recognized as a promising prognostic marker in cancer biology.

Accumulating evidence has demonstrated that B7-H4 promotes breast tumor progression by inducing T cell exhaustion [47], which underscores its critical role in mediating negative immune regulation within the tumor microenvironment. Pharmacologic inhibition of glycosylated B7-H4 has been reported to reduce cell surface expression of B7-H4 protein on breast cancer cells, thereby effectively abrogating the immunosuppressive effects induced by B7-H4 [29]. The ongoing clinical development of B7-H4-targeted agents, including AstraZeneca’s antibody-drug conjugate (ADC) AZD8205 and GSK’s ADC GSK5733584, further supports the translational relevance of B7-H4 as a therapeutic target in solid tumors. The clinical activity observed in B7-H4-expressing tumors highlights the importance of understanding the biological functions of B7-H4 and its role in tumor immune regulation [48–50].

B7-H4 protein is preferentially overexpressed in tumor cells across various human cancer tissues rather than most healthy tissues. Such an expression pattern differs from other B7-CD28 family members. For instance, B7.1, B7.2, CTLA-4, PD-1, and PD-L1 are expressed on antigen-presenting cells or resting T cells, or they are induced after stimulation. In contrast, B7-H4 exhibits a more extensive expression profile in human cancers. Structurally, the human B7-H4 IgV domain adopts a typical IgV organization, as revealed by crystal structure studies [51], whereas the structure of its IgC domain remains unresolved. For B7-CD28 family members, the receptor-binding interface is generally located in the IgV domain. However, in this study, we found it is the IgC domain of B7-H4 that interacts with Gal-9, not the IgV domain.

Even though our findings do not rule out the possibility that the IgV domain of B7-H4 binds to an as-yet unidentified receptor involved in immune-regulating functions—or that it may potentially interact with such a receptor through its IgV domain—our results, including the lack of an observable immune cell phenotype and the absence of an impact on anti-tumor responses against tumor challenge in B7-H4 deficiency in mice compared to WT mice, at least partially align with prior research [52,53]. These studies indicate that the impact of B7-H4 deficiency is minimal in mice on both C57BL/6 and BALB/c backgrounds. Similar results were observed in cancer models: B7-H4 KO in BALB/c mice challenged with mammary carcinoma cells (4T1) showed no differences in primary tumor growth [54]. However, in the 4T1 metastatic model, B7-H4 KO BALB/c mice exhibited a reduced lung metastasis burden [53]. Taken together, we surmise that B7-H4 likely plays a redundant and/or context-dependent role in immune regulation.

The context-dependent role for B7-H4 in mice has also been highlighted in other disease models observed in B7-H4 KO mice under conditions of additional challenges. In a Streptococcus pneumoniae infection model, B7-H4 deficient mice exhibited reduced disease severity, increased activation of CD4^+^ and CD8^+^ T cells in the lungs [55], suggesting a role for B7-H4 in suppressing T cell immunity. In autoimmune models, B7-H4 deficiency was associated with worsened disease outcomes [56]. For example, severe experimental autoimmune encephalomyelitis was marked by increased Th1 and Th17 responses in pancreatic islets and the central nervous system. In a kidney disease model, B7-H4 deficiency led to severe renal injury, enhanced humoral responses, and polarization of inflammatory macrophages [57]. These findings suggest that B7-H4 plays a regulatory role in immune responses across both infection and autoimmunity. However, whether these effects are mediated through an unidentified receptor interacting with its IgV domain or through Gal-9 binding to its IgC domain remains unclear.

Additionally, given our observation that Gal-9 interacts with multiple glycosylated protein partners, enabling context-dependent regulation, it is likely that B7-H4 functions as one of several negative co-signaling molecules that collectively fine-tune T cell-mediated immune responses within this context. Specifically, in this study, we found that several immune receptors, including CD28, 2B4, CD226, and SLAMF1, bind to Gal-9 at levels comparable to PD-1, TIM-3, and B7-H4. CD28 interacts with Gal-9 in a manner similar to its interaction with TIM-3, with the binding primarily mediated by the C-CRD domain of Gal-9, whereas the N-CRD of Gal-9 interacts with B7-H4. Other B7 family members, such as B7-H2, B7.1, B7-DC, and B7.2, were also found to bind to Gal-9 in our study. Previous studies also reported other Gal-9 binding partners, including TIM-3, PD-1, CD44, CD40, 4–1BB, death receptor 3 (DR3), V-domain Ig containing suppressor of T cell activation (VISTA), TLR-4, CD44, Dectin-1, Dectin-2, and ERMAP [25,58]. Given such a complex interaction network surrounding Gal-9, it is unsurprising that B7-H4 KO alone in mice does not result in immune cell phenotypes similar to those observed in Gal-9 KO mice.

Gal-9, a tandem-repeat type galectin consisting of two homologous but distinct CRDs (N-CRD and C-CRD), is expressed in almost all organs and plays a role in a variety of physiological functions, including cell growth, differentiation, adhesion, and cell death [59]. Its cellular location is often intracellular but can also be extracellular at the cell surface or within the extracellular matrix [21,31]. Within the immune system, Gal-9 is widely expressed in immune cells, with accumulating evidence demonstrating it acts as a pleiotropic immune modulator. As mentioned above, Gal-9 interacts with multiple glycosylated immune cell surface receptors and B7 family members, including both previously known ones and the new ones identified in this study, highlighting the complexity of its role in immune regulation. The variable expression of these receptors across cell types likely influences Gal-9 signaling outcomes. Additionally, Gal-9’s ability to form lattices, such as TIM-3-Gal-9-PD-1 [24], adds another layer of possible regulation mechanism depending on the co-expression of other receptors.

Galectins-mediated immune cell activation (e.g., Gal-3) is a multi-step process governed by glycan recognition, CLIC-dependent endocytosis [60,61], and Rab6/Syntaxin-16-dependent retrograde transport to the Golgi-TGN enables glycan remodeling and polarized delivery of signaling and adhesion molecules to the immune synapse [62], where they amplify TCR signaling and stabilize synapse architecture. Moreover, the discovery of cis-B7:CD28 signaling redefines CD28 activation, revealing a cell-autonomous mechanism that sustains T cell function. This molecular mechanism centers on membrane curvature-dependent cis-interactions, PI3K-SNX9-driven membrane remodeling, and PKCθ-mediated signal amplification [63]. Together, these mechanisms suggest a potential layer of regulation in Gal-9-induced CD28 activation, wherein glycan-dependent trafficking networks (glycan recognition of T cell surface molecules to initiate their clustering, membrane remodeling, the endocytosis and retrograde transport to the Golgi-TGN, or an integral component of immune synapse formation) orchestrate the spatial and temporal control of T cell biology.

Phosphorylation of Y191 on CD28 promotes the recruitment of phosphoinositide 3-kinase (PI3K) to CD28 [64,65]. However, Jurkat T cells harbor a defect in the tumor suppressor PTEN, a phosphatase that negatively regulates the PI3K/AKT signaling pathway [66]. PTEN deficiency leads to accumulation of phosphatidylinositol 3,4,5-trisphosphate (PIP_3_) and constitutive phosphorylation of AKT at S473, even in unstimulated cells [66,67]. Thus, the elevated basal PI3K/AKT signaling activity in Jurkat T cells may blunt the detection of Gal-9-induced AKT phosphorylation [68]. Consistent with these observations, we observed high baseline phosphorylation of AKT at S473 in Jurkat T cells, a pattern distinct from that observed in MOLT-4 cells and primary T cells. By contrast, basal phosphorylation of CD28 at Y191 was readily detectable in MOLT-4 and primary T cells but was low to undetectable in Jurkat T cells, allowing clearer detection of Gal-9-induced changes in in CD28 signaling. Together, these findings support the use of multiple T-cell models and the simultaneous assessment of signaling events upstream and downstream of AKT when evaluating CD28 signaling pathways in human T cells.

In this study, since B7-H4 was identified as a binding partner of Gal-9, we further investigated B7-H4’s effect on Gal-9’s activity. Given the aforementioned complex and multifaceted nature of Gal-9’s activity, we specifically focused on its well-known dual immune-regulatory roles: stimulating or suppressing T cell activity, depending on its cellular location and the conditions [16,31,32]. Intracellular Gal-9 has been shown to translocate to the membrane during the immune synapse formation upon stimulation, and Gal-9 deficiency in T cells impaired the phosphorylation of proximal TCR signaling proteins, suggesting that Gal-9 may participate in the modulation of TCR signals [16]. Notably, we found that Gal-9 binds to CD28, a pivotal co-stimulatory receptor that enhances TCR signaling. Furthermore, we observed that Gal-9 binding to CD28 on T cells stimulated CD28 signaling activation in a manner similar to B7.1. Gal-9 also exhibited additive effects with B7.1 in enhancing this activation. Additionally, B7-H4, by binding to Gal-9, interferes with this signaling process and inhibits Gal-9-induced CD28 activation in T cells in an IgC domain and glycosylation-dependent manner. Our findings provide at least one mechanism that may explain Gal-9-mediated T cell stimulation.

Exogenous soluble Gal-9 is known for its role in inducing T cell apoptosis [23,33,36], suggesting that extracellular Gal-9 *in vivo* may also exert this effect, triggering apoptosis in T cells. Supporting this notion, we found that Gal-9 KO mice exhibited a significant increase in the proportion of splenic CD4^+^ T cells. *In vitro*, we also found that soluble Gal-9-induced T cell death in primary T cells and other T cell lines, and B7-H4 reduced this process by binding to Gal-9 in a glycosylation-dependent manner. Research has been conducted to elucidate the mechanisms underlying Gal-9-induced T cell death, with TIM-3- or VISTA-mediated mechanisms being reported [23,69]. The finding that B7-H4 inhibits Gal-9-induced T cell death appears contradictory to B7-H4’s known inhibitory immune-regulating role. However, this effect may resemble the process in which PD-1 binding to Gal-9, reducing TIM-3-dependent T cell death [24] and thereby contributing to the persistence of a specific subset of exhausted T cells. Alternatively, B7-H4 may compete with TIM-3 or other Gal-9 binding partners on T cells to attenuate Gal-9-induced T cell death, reflecting a complex network of immune regulation that relies on the balanced interplay of activating and suppressing signals, as well as spatial and temporal dynamics.

Emerging evidence suggests that the N- and C-terminal CRDs of Gal-9 contribute differently to its immunomodulatory functions. Li et al. demonstrated that, although both CRDs can induce T cell death, the C-CRD is more potent in this activity, whereas the N-CRD more effectively activates dendritic cells and induces IκBα degradation [59]. Recent HIV studies further revealed opposing functions of the two CRDs, with the N-CRD promoting Treg development and the C-CRD inhibiting Treg induction [70]. Our findings that B7-H4 preferentially engages the N-CRD (R65-dependent), whereas TIM-3 interacts with the C-CRD, suggest that distinct downstream signaling outcomes may result from CRD-specific ligand engagement. This functional specialization may enable Gal-9 to integrate diverse immune signals and modulate T-cell responses in a context-dependent manner.

Based on previous studies and our current data, we hypothesize the following potential binding modalities at the Antigen-presenting cells (APC)–T cell immune synapse interface: (1) On APC within the immune synapse, B7.1 mediates trans-B7.1:CD28 binding, which promotes T cell activation via the CD28 signaling pathway [71]. Concomitantly, Gal-9 engages in trans-Gal-9:TIM-3 binding at the synapse, thereby inducing T cell death [23,24]. (2) On APC within the immune synapse, the APC-expressed ligand B7-H4 preferentially forms trans-B7-H4:Gal-9 interactions, rather than trans-Gal-9:CD28 or trans-Gal-9:TIM-3 binding. Notably, B7-H4 attenuates Gal-9-mediated CD28 activation and T cell death. (3) On T cells within the immune synapse, B7.1 or Gal-9 is expressed, enabling cis-B7.1:CD28 [63] or cis-Gal-9:CD28 binding. The ligation of Gal-9 to the co-stimulatory receptor CD28 may potentiate Gal-9-induced CD28 activation. (4) On T cells within the immune synapse, the co-inhibitory receptor PD-1 and TIM-3 form cis-PD-1:Gal-9:TIM-3 complexes. Ligation of the PD-1:Gal-9 complex to TIM-3 may attenuate TIM-3-mediated T cell death [24]. Future studies using advanced imaging techniques such as confocal microscopy or FRET will be valuable to visualize B7-H4-Gal-9 colocalization and direct interaction at the APC–T cell immune synapse interface, further confirming the physiological relevance of this interaction.

We acknowledge the limitations of our current study. Although our data demonstrate specific binding between B7-H4 and Gal-9, future studies systematically comparing B7-H4 interactions with other galectin family members, such as Gal-1, Gal-3, Gal-4, and Gal-8, will be important to further validate the specificity of this interaction and determine whether other galectins contribute to B7-H4-mediated immune regulation. Gal-9 stimulates pAKT signaling through trans-interaction with the co-stimulatory receptor CD28 on T cells. Beyond CD28, whether and how other Gal-9-binding immune checkpoint receptors modulate Gal-9 activity remain unclear. In addition, the molecular mechanism through which Gal-9 induces T-cell death remains to be elucidated. Gal-9-induced T-cell death may involve apoptotic signaling and/or rapid membrane-permeability changes mediated by one or more other glycosylated surface receptors, but the precise receptor and downstream death pathway remain to be defined.

In summary, our study identifies Gal-9 as a binding partner of B7-H4, along with a number of previously unknown Gal-9 binding partners. Gal-9 interacts with the co-stimulatory receptor CD28 on human T cells, leading to enhanced activation of downstream signaling. Additionally, Gal-9 induces T cell death *in vitro*, a finding supported by *in vivo* observations where Gal-9 deficiency in mice drives a significant increase in the proportion of splenic CD4^+^ T cells, underscoring the role of Gal-9 in T cell death. B7-H4 binding to Gal-9 reduces these Gal-9’s activities, offering novel insights into the molecular mechanism by which B7-H4 contributes to the T cell-mediated immune regulations. Nevertheless, the physiological and pathological relevance of these findings remains to be fully elucidated and warrants further investigation.

## Supporting information

S1 Fig The identification of B7-H4 binding proteins. **(A-B)** Flow cytometry analysis of B7-H4 binding to naïve and activated T cells. hB7-H4-Fc binding to activated human T cells (A), and mB7-H4-Fc binding to naïve or cell-cultured OVA-activated OT-1 T cells (B). Histogram overlays show the isotype control group human CD147-Fc (shaded gray), and potential binding signals stained by hB7-H4-Fc or mB7-H4-Fc (black line). **(C-D)** Flow cytometry analysis of mB7-H4-Fc binding to adoptive-transfer cells (C) and host CD45.1^+^ cells (D) in the C57BL/6J (CD45.1^+^, CD45.2^-^) mice transferred with EG7 tumor cells and OT-1 T cells on day 7. Two-dimensional plots show the gating strategy to identify each immune cell lineage (top panel) and the blue arrows represent the chronology for the gating strategy. Histogram overlays represent mB7-H4-Fc binding (black line) and isotype control binding (shaded gray) to each immune cell lineage (bottom panel). These Fc tag proteins were tested at 10 µg/mL. **(E)** Immunoprecipitation using mB7-H4-Bio. Cell lysates from 4T1 tumor cells isolated from 4T1 tumor-bearing mice, EG7 tumor cells isolated from EG7 tumor-bearing mice, or CD45.1^+^ peritoneal immune cells isolated from the adoptive-transfer-model mice (as described in Methods and Fig 1B) were precipitated using mB7-H4-Bio. The arrow indicates the ~ 35 kDa band precipitated by mB7-H4-Bio in CD45.1^+^ peritoneal immune cell lysates but not in 4T1 or EG7 tumor cell lysates. **(F)** The protein sequence of mouse Gal-9 (UniProt O08573-2, residues 1–322, 36.5 kDa). The seven peptide sequences in red represent those identified by LC-MS/MS-based proteomic analysis of the 35 kDa protein band.
(TIFF)

S2 Fig Characterization of the binding between de-glycosylated B7-H4 mutants and Gal-9. **(A)** A schematic of the human B7-H4 ectodomains and their glycosylation sites. Seven glycosylation sites are marked by asterisks, and the sites required for the interaction with Gal-9 are highlighted in red. **(B)** SDS-PAGE analysis of glycosylated hB7-H4-Fc and its de-glycosylated forms or single-site mutants. **(C)** SPR analysis of Gal-9 binding to B7-H4 glycosylation mutants. To test its binding, 1.2 µg/mL Gal-9 was flowed through a Protein A/G-coated chip immobilized with individual B7-H4 mutants.
(TIFF)

S3 Fig Gal-9 decreases T cell viability. **(A)** Two-dimensional plots show the gating strategy to identify each immune cell lineage and the blue arrows indicate the gating sequence. **(B)** Gal-9 reduces the cell viability of human PBMCs. Flow cytometry dot plots show the proportion of live human PBMCs after treatment with 8 µg/mL Gal-9 for 0.5 h. **(C-E)** Gal-9 reduces the immune cell viability in PBMCs. Flow cytometry dot plots show the proportions of dead-cell dye-negative live CD3^+^ T cells (C), CD19^+^ B cells (D), and CD14^+^ myeloid cells (E) within the corresponding parent immune-cell gates defined in Panel A. **(F-G)** Gal-9 reduces the cell viability of primary human T cells. Flow cytometry dot plots show the proportions of live cell (7-AAD negative) within serum-starved primary T cells after treatment with 8 µg/mL Gal-9 for 0.5 h (F) or 18 h (G). Statistical significance was determined by unpaired t-tests (Panels B-G), to assess live T cells treated with Gal-9 compared to those untreated. For each panel (B-G), data from three independent replicates (mean ± s.e.m.) are shown on the right.
(TIFF)

S4 Fig Gal-9 interacts with multiple immune checkpoint receptors. **(A)** SPR binding kinetics between Gal-9 and the ectodomains of ten immune checkpoint receptors. The SPR analysis used the same method as mentioned in Fig 4A. **(B)** Flow cytometry analysis of the binding activities between human 2B4, CD226, and SLAMF1 and 293T cells transiently overexpressing either WT Gal-9 or single-site mutant Gal-9 R65A. The analysis used the same method as Fig 4B. **(C)** Representative immunoblots of pAKT and AKT signaling in MOLT-4 T cells. The MOLT-4 T cells were stimulated with 6 µg/mL Gal-9 in the presence or absence of 8 µg/mL hB7-H4-Fc. Three independent experiments were performed for each condition by western blotting. **(D-E)** Quantification of pAKT band intensity in MOLT-4 T cells stimulated with Gal-9 in the absence (D) or presence (E) of 8 µg/mL hB7-H4-Fc. Band intensities were quantified using ImageJ and normalized to total AKT. Fold change (FC) relative to the AKT loading control was analyzed using paired t tests. **(F-G)** Flow cytometry analysis of T-cell viability in MOLT-4 T cells treated with Gal-9 and hB7-H4-Fc. Statistical analysis of MOLT-4 T-cell viability across all treatment groups is shown in panel G. Three independent replicates were performed for each condition. One-way ANOVA was used to compare T-cell viability among the three treatment conditions. Data are presented as mean  ±  s.e.m.
(TIFF)

S5 Fig Gal-9 interacts with B7 family members. **(A)** SPR binding kinetics between Gal-9 and the ectodomains of seven members in the B7 family. The SPR analysis used the same method as Fig 4A. **(B)** Flow cytometry analysis of the binding between the B7 family members and 293T cells transiently overexpressing WT Gal-9. Flow cytometry analysis used the same method as mentioned in Fig 4B. **(C)** 8 µg/mL hB7-H4-Fc, hB7-H3-Fc, and hB7-H1-Fc modulated the stimulation of pAKT signaling activity in primary T cells which were stimulated by 6 µg/mL Gal-9 for a 0.5 h incubation by western blotting (top panel). Fold change (FC) of band intensity over AKT loading control was quantified by ImageJ and GraphPad Prism (bottom panel). **(D)** 8 µg/mL hB7-H4-Fc, hB7-H3-Fc, and hB7-H1-Fc modulated the proportion of live cells (7-AAD negative) within primary T cells treated with 6 µg/mL Gal-9 for a 0.5 h incubation as determined by flow cytometry analysis of T cell viability.
(TIFF)

S6 Fig Genotypic and phenotypic characterization of splenic immune cells in Gal-9 KO mice and B7-H4 KO mice. **(A-B)** Schematic diagram of Gal-9 KO (A) and B7-H4 KO (B) design in WT C57BL/6J mice. For Gal-9 KO, genotype primers, F1 and R1, are designed to identify WT allele containing intact mouse Gal-9 gene—the Exon2 (89 bp) of mouse Gal-9 gene, while F2 and R2 are designed to identify Gal-9 KO allele. For B7-H4 KO, genotype primers, F3 and R3, are designed to identify WT allele containing intact mouse B7-H4 gene, while F4 and R4 are designed to identify B7-H4 KO allele lacking the whole KO fragment (including Exon3, encoding for mouse B7-H4 IgV domain; and Exon4, encoding for mouse B7-H4 IgC domain). **(C-D)** Genotyping results of Gal-9 KO (C) and B7-H4 KO (D) newborn mice. The PCR products, ① and ②, correspond to the target genes amplified using the genotype primers as described above in Panel A; the PCR products, ③ and ④, correspond to the target genes amplified using the genotype primers as described above in Panel B. **(E)** Genotyping of B7-H4 KO and B7-H4/Gal-9 DKO newborn mice. The PCR products correspond to the target genes using all four pairs of genotype primers as described in Panels A-B. **(F)** Flow cytometry analysis of CD45^+^ live or dead splenic cells in Gal-9 KO mice compared to those in WT mice. These data are from the same experiment as described in Figs 6A-6B. Representative dot plots from one mouse are shown on the left. The data from all mice (presented as mean  ±  s.e.m.), along with the statistical analysis of splenic CD45^+^ dead cells in Gal-9 KO mice compared to WT mice, are presented on the right (n = 8 mice for WT group, n = 7 mice for Gal-9 KO group). **(G)** The data from all mice are shown in Panel F, along with the statistical analysis of splenic B cells, CD11b^+^ cells or NK cells in Gal-9 KO mice compared to WT mice. The unpaired t-test was used for two-group statistical comparisons as described in Panels F-G.
(TIFF)

S7 Fig MC38 tumor growth exhibits no difference between B7-H4 KO mice and Gal-9 KO mice. **(A)** Flow cytometry analysis of B7-H4 expression on MC38-mB7-H4 stable cells. Histogram overlays show the isotype control antibody (shaded gray) and mB7-H4 expression detected using anti-mB7-H4 antibody (black line). **(B)** MC38 tumor growth in WT, Gal-9 KO, and B7-H4/Gal-9 DKO mice. The data shown on the left is from one experiment (n = 3 mice per group), while the data shown on the right is from another independent experiment, n = 5 mice per group, except for the WT C57BL/6J mice (n = 4). All data are presented as means ± s.e.m.
(TIFF)

S1 Data Supporting dataset. (XLSX)
